# XIST dampens X chromosome activity in a SPEN-dependent manner during early human development

**DOI:** 10.1038/s41594-024-01325-3

**Published:** 2024-06-04

**Authors:** Charbel Alfeghaly, Gaël Castel, Emmanuel Cazottes, Madeleine Moscatelli, Eva Moinard, Miguel Casanova, Juliette Boni, Kasturi Mahadik, Jenna Lammers, Thomas Freour, Louis Chauviere, Carla Piqueras, Ruben Boers, Joachim Boers, Joost Gribnau, Laurent David, Jean-François Ouimette, Claire Rougeulle

**Affiliations:** 1 Epigenetics and Cell Fate, CNRS, Université Paris Cité, Paris, France; 2 Center for Research in Transplantation and Translational Immunology (CR2TI), CHU Nantes, Inserm, Nantes Université, Nantes, France; 3 Service de Biologie de la Reproduction, CHU Nantes, Nantes Université, Nantes, France; 4 Department of Developmental Biology, Erasmus University Medical Center, Rotterdam, Netherlands; 5 BioCore, CNRS, CHU Nantes, Inserm, Nantes Université, Nantes, France

**Keywords:** Dosage compensation, Pluripotent stem cells, Pluripotency, Chromatin, Long non-coding RNAs

## Abstract

XIST (X-inactive specific transcript) long noncoding RNA (lncRNA) is responsible for X chromosome inactivation (XCI) in placental mammals, yet it accumulates on both X chromosomes in human female preimplantation embryos without triggering X chromosome silencing. The XACT (X-active coating transcript) lncRNA coaccumulates with XIST on active X chromosomes and may antagonize XIST function. Here, we used human embryonic stem cells in a naive state of pluripotency to assess the function of XIST and XACT in shaping the X chromosome chromatin and transcriptional landscapes during preimplantation development. We show that XIST triggers the deposition of polycomb-mediated repressive histone modifications and dampens the transcription of most X-linked genes in a SPEN-dependent manner, while XACT deficiency does not significantly affect XIST activity or X-linked gene expression. Our study demonstrates that XIST is functional before XCI, confirms the existence of a transient process of X chromosome dosage compensation and reveals that XCI and dampening rely on the same set of factors.

## Abstract (web summary)

Using naive human embryonic stem cells as a model for early embryogenesis, the authors report that the XIST (X-inactive specific transcript) long noncoding RNA recruits repressive histone marks and attenuates X chromosome expression before the establishment of X chromosome inactivation.

## Main

Male and female mammals differ genetically by the presence of sex chromosomes, with the latter carrying two X chromosomes and the former carrying one X and one Y chromosome. The human X chromosome is one of the seven largest chromosomes, harboring ~1,100 genes and 4% of the total number of protein-coding genes, both housekeeping and tissue specific. X chromosome dosage imbalance between male and female individuals is compensated for by the transcriptional inactivation of one of the two X chromosomes in females^1–3^. In mouse, X chromosome inactivation (XCI) is set up during early female embryogenesis, soon after zygotic genome activation^4–6^. Such timing suggests that functional compensation of X chromosome dosage is essential not only for cellular function and homeostasis in adults but also for early development, a hypothesis that is confirmed by the female embryonic lethality caused by improper XCI establishment^7,8^.

In contrast to rodents, primate female preimplantation development proceeds in the absence of XCI^9,10^. Strikingly, XIST (X-inactive specific transcript), which triggers XCI, is already expressed at these early stages in humans and XIST RNA decorates both active X chromosomes^10,11^. Xist RNA has been shown in the mouse to recruit a plethora of protein factors that induce a cascade of remodeling events leading to transcriptional silencing and chromatin reorganization of the inactive X chromosome^12,13^. One of these effectors is SPEN, which has a key role in the initiation and establishment of XCI^14,15^. SPEN binds Xist by its RNA recognition motifs (RRMs) and interacts, through its SPOC domain, with multiple factors, including the corepressors NCoR and SMRT, as well as the NuRD complex, to activate histone deacetylase 3 (HDAC3) and to promote transcriptional repression^14,15^. In addition, Xist indirectly recruits the noncanonical polycomb repressive complex (PRC) 1, leading to the accumulation of repressive histone modifications such as H2AK119Ub and PRC2-mediated H3K27me3, which are hallmarks of the inactive X chromosome^16–18^. PRC1 and SPEN have been shown to act in parallel in the repression of X-linked genes, while PRC2 appears mostly dispensable^19^.

The uncoupling between XIST expression and XCI establishment in human early development raises questions as to whether XIST is functional at this stage. The ability of XIST to recruit enzymatically active histone-modifying complexes is challenged by the reported lack of accumulation of repressive histone modifications on XIST-coated X chromosomes in human preimplantation embryos^10^. Additionally, whether XIST intervenes in other transcriptional compensation mechanisms that may operate during this specific time window is an open question. Indeed, X chromosome dampening (XCD), which corresponds to reduced X-linked gene expression from both active X chromosomes, has been reported to take place progressively during the first days of female embryogenesis^20^. Lastly, the presence of yet unknown factors preventing XIST from silencing the X chromosome remains to be determined.

We previously identified XACT (X-active coating transcript), a primate-specific long noncoding RNA (lncRNA) that is expressed and accumulates in cis on both active X chromosomes during early human development^11,21,22^. The expression of XACT is mainly restricted to pluripotent stages, suggesting a role for XACT in this cellular context^22^. Previous studies have shown that XIST and XACT RNAs occupy distinct nuclear territories in human preimplantation embryos and XACT expression in transgenic mouse cells influences XIST accumulation in cis^11^. This renders XACT an interesting candidate that might act as an XCI antagonist by affecting XIST expression, localization or activity.

Here, we capitalized on female human embryonic stem cells (hES cells), which can be transitioned from a primed to naive state of pluripotency capturing post-XCI and pre-XCI status, respectively, to study X chromosome activity and dosage compensation mechanisms in early human development. Using constitutive and inducible loss-of-function (LOF) approaches, we demonstrate that XACT does not impact X-linked gene expression in naive hES cells, despite XIST being functional in this context. Indeed, we show that XIST is responsible for a reduction in most but not all X-linked gene transcription levels and for the accumulation of PRC-mediated repressive histone marks on active X chromosomes. However, we uncover a massive redistribution of XIST RNA and PRC-mediated histone modifications on dampened compared to inactive X chromosomes. We also report enrichment in H3K27me3 on X chromosomes in embryos, thus resolving a long-standing question in the field and reconciling in vitro models to ex vivo embryos. Lastly, we provide evidence that XIST interacts with SPEN before XCI and reveal a direct role of SPEN in XCD. Overall, our study demonstrates that a core set of factors triggers distinct dosage compensation mechanisms in a developmental stage-dependent manner.

## Results

### XIST attenuates X chromosome transcription in naive hES cells

**Fig. 1:**
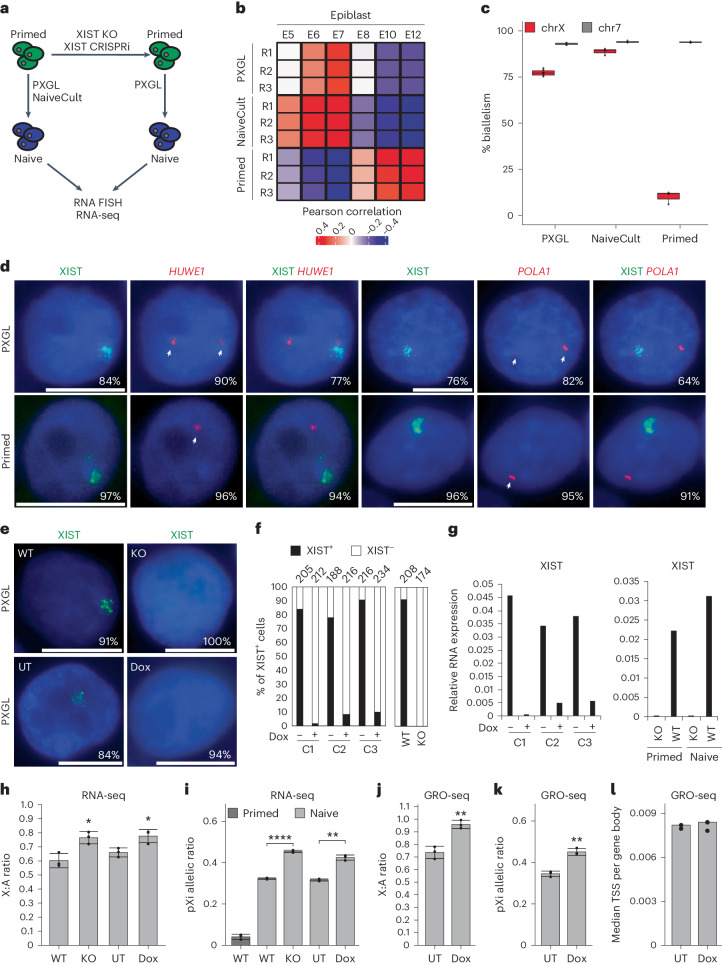
XIST attenuates X chromosome transcription in naive hES cells. **a**, Scheme of XIST depletion approach (CRISPR–Cas9 or CRISRPi) in primed H9 hES cells. **b**, Correlation heatmap generated using epiblast markers specific to each blastocyst stage from human preimplantation and postimplantation embryo single-cell RNA (scRNA)-seq data^20,25^. Numbers correspond to the embryonic days (E5 to E12). **c**, Percentage of biallelically expressed SNPs from X chromosome and chromosome 7 in naive (NaiveCult, *n* = 3; PXGL, *n* = 6) and primed (*n* = 3) H9 hES cells obtained from RNA-seq data. Box plots represent the median (center), first and third quartiles (hinges) and ±1.5 interquartile range (IQR; whiskers). **d**, Representative RNA FISH images for XIST and two X-linked genes (*HUWE1* and *POLA1*) in naive (PXGL) and primed H9 hES cells. Percentages of cells displaying the representative pattern are indicated. Scale bar = 10 µm. **e**, Representative RNA FISH images for XIST in naive H9 hES cells with a stable or Dox-inducible XIST repression. Percentages of cells displaying the representative pattern are indicated. Scale bar = 10 µm. **f**, Quantification of RNA FISH data showing the percentage of cells with XIST signal in XIST WT, XIST KO and three XIST CRISPRi naive H9 clones (C1–C3) treated or not with Dox. **g**, RT–qPCR analysis of XIST expression in naive XIST CRISPRi clones with or without Dox treatment (left) and in XIST WT and KO naive and primed H9 hES cells (right). Values are normalized to β-actin. **h**, X:A ratio from RNA-seq data of XIST WT, KO, UT CRISPRi and Dox-treated (Dox) CRISPRi naive H9 cells (*n* = 3). Data are presented as the mean values ± s.d. of replicates. **i**, pXi allelic ratio from RNA-seq data of primed (WT) and naive (XIST WT, KO, UT CRISPRi and Dox-treated CRISPRi) H9 hES cells (*n* = 3). Data are presented as the mean values ± s.d. of replicates. **j**, X:A ratio from fastGRO-seq data of XIST UT and Dox-treated CRISPRi naive H9 cells (*n* = 3). Data are presented as the mean values ± s.d. of replicates. **k**, pXi allelic ratio from fastGRO-seq data of XIST UT and Dox-treated CRISPRi naive H9 cells (*n* = 3). Data are presented as the mean values ± s.d. of replicates. **l**, Ratio of the median coverage of reads on the TSS and gene body from fastGRO-seq data from XIST UT (*n* = 2) and Dox-treated (*n* = 3) CRISPRi naive H9 hES cells. Unless otherwise specified, *P* values were calculated by a two-sided unpaired *t*-test. Levels of significance: not significant (NS; *P* ≥ 0.05), *P < 0.05, **P < 0.01, ***P < 0.001 and ****P < 0.0001. Source data

**Extended Data Fig. 1:**
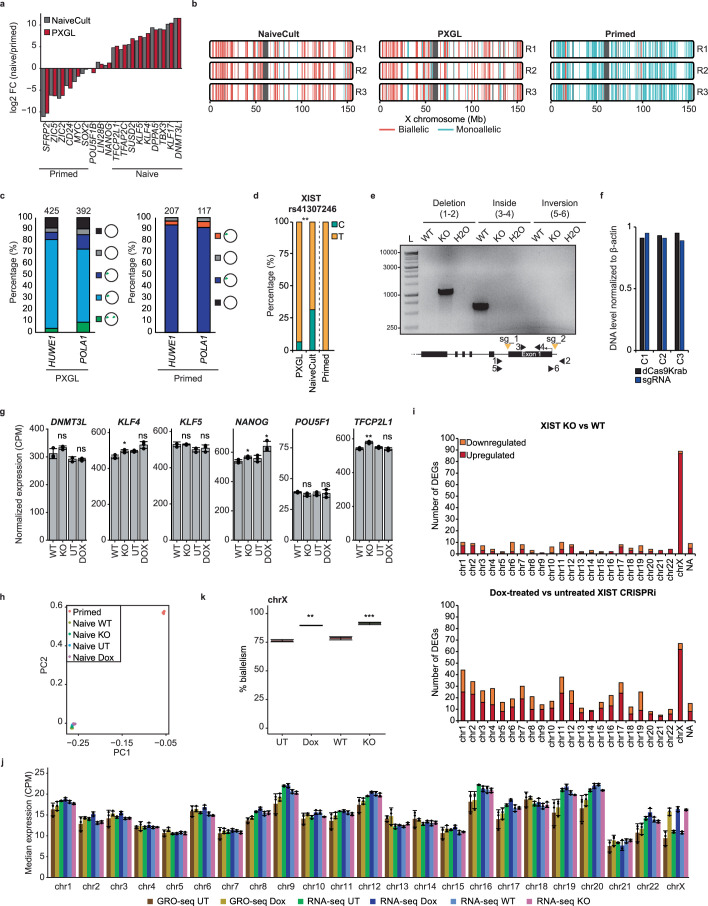
Characterization of the X chromosome status in naïve XIST KO and XIST CRISPRi clones. **a**, Bar plot comparing log2 FC in RNA-seq data between naïve (NaïveCult and PXGL) and primed H9 hESCs (n = 3). Core pluripotency factors and state-specific markers are highlighted. **b**, X chromosome map showing positions and allelic status of expressed SNPs in naïve (NaiveCult and PXGL) and primed H9 hESCs. **c**, RNA-FISH data quantification showing percentage of cells with monoallelic or biallelic XIST (green) and/or X-linked genes (HUWE1 and POLA1; red) signals in naïve (PXGL) and primed H9 hESCs. **d**, Percentage of XIST allelic expression in naïve (NaiveCult and PXGL) and primed H9 hESCs, based from RNA-seq data. **e**, Top: Gel images of PCR products from WT and XIST-targeted primed H9 hESCs using various screening primers on genomic DNA. Bottom: Schematic of the XIST deletion approach indicating CRISPR sgRNA locations (yellow triangles) and screening primer positions (black arrows). **f**, Copy number analysis of dCas9Krab and sgRNA insertion in the 3 XIST CRISPRi clones (C1-C3) using qPCR on genomic DNA. Values are normalized to β-actin. **g**, Expression levels of core and naïve specific pluripotency factors in naïve H9 WT, XIST KO and XIST CRISPRi clones untreated (UT) or treated with Dox, from RNA-seq data (n = 3). Data are presented as mean values +/- SD of replicates. **h**, PCA plot displaying the first two principal components of the RNA-seq data from naïve (WT, XIST KO, XIST CRISPRi UT and dox-treated) and WT primed H9 hESCs. **i**, Number of DEGs upon XIST KO (top) and XIST CRISPRi (bottom) repression in naïve H9 cells. **j**, Median expression values per chromosome in naïve WT, XIST KO and XIST CRISPRi clones UT or treated with Dox, obtained from RNA-seq and fastGRO-seq data (n = 3). Data are presented as mean values +/- SD of replicates. **k**, Percentage of biallelically expressed X chromosome SNPs in naïve H9 WT, XIST KO, and XIST CRISPRi clones (UT or Dox-treated, n = 3) based on RNA-seq data. Boxplots represent the median (center) first and third quartile (hinges) and ±1.5 × IQR (whiskers). Unless otherwise specified, p-values were calculated by two-sided unpaired *t*-test. Levels of significance: ns≥0.05; * < 0.05; ** < 0.01; *** < 0.001; **** < 0.0001. Source data

To study the X chromosome status in early development and to probe the function of XIST before XCI establishment, we took advantage of primed to naive reprogramming of H9 hES cells using NaiveCult (StemCell Technologies) and PXGL media (Fig. 1a)^23,24^. We validated through transcriptomic analyses the identity of chemically reprogrammed H9 cells, which express naive-specific pluripotency markers (Fig. 1b and Extended Data Fig. 1a) and display transcriptomic signatures of preimplantation epiblast E6 and E7 cells^20,25^, while primed H9 hES cells are more similar to cells of postimplantation epiblasts (Fig. 1b). We also verified the X chromosome activity status using a list of X-linked informative single-nucleotide polymorphisms (SNPs) distributed over approximately 140 genes (Supplementary Table 1). This confirmed that X chromosome reactivation occurred, with biallelic expression of the vast majority of tested X-linked SNPs (87% and 75% in naive H9 hES cells reprogrammed using NaiveCult and PXGL, respectively, compared to 12% in primed hES cells; Fig. 1c and Extended Data Fig. 1b). X chromosome reactivation was confirmed at the single-cell level using RNA fluorescent in situ hybridization (FISH) for the X-linked genes *HUWE1* and *POLA1* (Fig. 1d and Extended Data Fig. 1c). Consistent with previous reports^11,26^, XIST was expressed and mostly accumulated on one of the two X chromosomes in naive H9 cells (Fig. 1d and Extended Data Fig. 1c). Allelic analysis showed that XIST expression was mainly from the same X chromosome in naive and primed hES cells (Extended Data Fig. 1d).

We generated XIST LOF systems based on (1) clustered regularly interspaced short palindromic repeats (CRISPR)–Cas9-mediated genome editing and (2) a doxycycline (Dox)-inducible CRISPR–dCas9 (dead Cas9) strategy to deplete XIST expression in H9 hES cells in a constitutive or inducible manner, respectively (Fig. 1a). One stable homozygous XIST knockout (KO) (Extended Data Fig. 1e) and three independent XIST CRISPR interference (CRISPRi) primed hES cell clones (Extended Data Fig. 1f) were obtained and converted to the naive state using the PXGL culture regimen.

XIST was efficiently repressed in naive XIST KO and Dox-treated CRISPRi clones, as measured by RNA FISH and reverse transcription (RT)–qPCR (Fig. 1e–g). Of note, the ability to derive XIST KO naive H9 cells with normal expression of naive-specific and core pluripotency factors indicates that XIST is not required for primed to naive resetting (Extended Data Fig. 1g). Principal component analysis (PCA) of RNA sequencing (RNA-seq) data generated from stable XIST KO and inducible XIST CRISPRi clones revealed that naive XIST^+^ (wild-type (WT) and untreated (UT) CRISPRi clones) and XIST^−^ (KO and Dox-treated CRISPRi clones) hES cells were grouped together and clustered away from primed hES cells, indicating that the principal variation is because of the cellular state and loss of XIST does not alter the global naive transcriptome (Extended Data Fig. 1h). Nevertheless, differential expression analysis revealed 216 (156 upregulated and 60 downregulated) and 546 (351 upregulated and 195 downregulated; log_2_FC (fold change) > |1| and FDR (false discovery rate) < 0.05) differentially expressed genes (DEGs) upon stable or inducible XIST repression, respectively (Extended Data Fig. 1i and Supplementary Tables 2 and 3). Of note, the higher number of DEGs upon Dox-induced XIST repression compared to XIST KO was likely because of a Dox effect^27^. Importantly, the majority of DEGs were on the X chromosome and upregulated, consistent with XISTʼs repressive activity (Extended Data Fig. 1i).

We next probed the impact of XIST loss on global X chromosome activity in naive hES cells by measuring the X:A ratio, which was around 1.3-fold higher in XIST KO and Dox-treated CRISPRi clones compared to WT and UT CRISPRi clones (Fig. 1h). This increase was not because of changes in global autosomal gene expression levels, which remained constant (Extended Data Fig. 1j). This indicates a global upregulation of the X chromosome expression in the absence of XIST, which also translates to a slight increase in the percentage of biallelically detected X-linked SNPs in naive XIST^−^ cells compared to XIST^+^ cells (Extended Data Fig. 1k). The level of increase (1.3-fold) was compatible with XIST being expressed from and regulating only one X chromosome. This suggests that loss of XIST led to higher expression from the formerly XIST-coated X chromosome in naive hES cells.

We took advantage of the fact that XIST was expressed from only one of the two active X chromosomes in naive hES cells (the previous Xi (pXi), Extended Data Fig. 1d) to confirm that the underlying effects were directly linked to the loss of XIST. As all primed H9 hES cells had the same inactive X, we could assign the reads overlapping an SNP to either the pXi or previous Xa (pXa) in naive hES cells and calculate a pXi allelic ratio. As expected, this ratio was 0.04 in primed hES cells, indicating that most reads were assigned to the active X chromosome (Fig. 1i). In contrast, XIST WT and UT CRISPRi naive hES cells displayed a pXi allelic ratio of 0.35, indicating that both X chromosomes were active, yet the pXi was lower expressed compared to the pXa (Fig. 1i). The pXi allelic ratio was significantly increased in the absence of XIST to reach 0.5, indicating equal expression from both X chromosomes (Fig. 1i). This demonstrates that XIST accumulation attenuated X chromosome expression in cis in naive hES cells.

To probe whether XIST-mediated dampening impacts X-linked gene expression at the transcriptional or post-transcriptional level, we measured nascent transcription using the low-input fast global run-on (fastGRO)-seq approach^28^. We observed an increase in the X:A and pXi allelic ratios in the absence of XIST similar to that observed by bulk RNA-seq (Fig. 1j, k). This shows that XIST regulates the ongoing transcription of X-linked genes in naive hES cells. As we found no significant difference in the promoter-proximal pausing ratio between UT and Dox-treated CRISPRi XIST clones (Fig. 1l), we concluded that XIST-mediated attenuation acts at the level of transcription initiation. Altogether, our data show that XIST accumulation attenuates X chromosome transcription in naive hES cells.

### XACT does not control X chromosome activity in naive hES cells

**Fig. 2:**
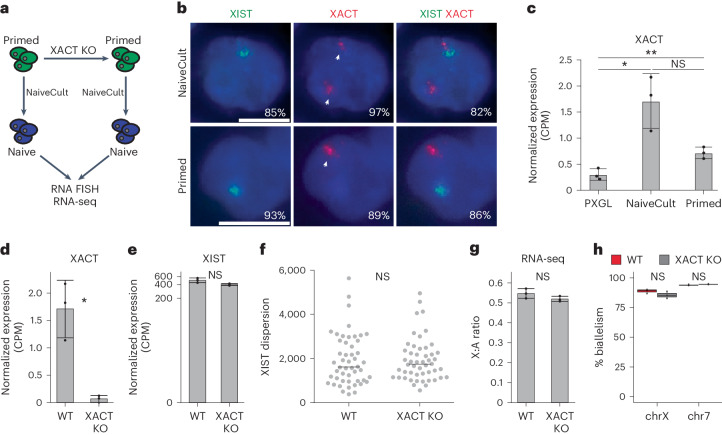
XACT does not control X chromosome activity in naive hES cells. **a**, Scheme of XACT deletion using CRISPR–Cas9 approach in primed H9 hES cells. Primed WT and XACT KO H9 cells were converted to the naive state using NaiveCult protocol. **b**, Representative RNA FISH images for XIST and XACT in naive (NaiveCult) and primed H9 hES cells. Percentages of cells displaying the representative pattern are indicated. Scale bar = 10 µm. **c**, XACT expression levels in naive (PXGL and NaiveCult) and primed H9 cells obtained from RNA-seq data (*n* = 3). Data are presented as the mean values ± s.d. of replicates. **d**, XACT expression level in XACT WT and KO naive hES cells obtained from RNA-seq data (*n* = 3). Data are presented as the mean values ± s.d. of replicates. **e**, XIST expression level in XACT WT and KO naive cells obtained from RNA-seq data (*n* = 3). Data are presented as the mean values ± s.d. of replicates. **f**, Distribution of the dispersion of XIST RNA FISH signal in XACT WT and KO naive cells (*n* = 50 cells per condition examined over one experiment). The horizontal line represents the median dispersion of each group; Wilcoxon *P* value ≥ 0.05 (NS). **g**, X:A ratio from RNA-seq data of WT and XACT KO naive H9 cells (*n* = 3). Data are presented as the mean values ± s.d. of replicates. **h**, Percentage of biallelically expressed SNPs from the X chromosome and chromosome 7 in XACT WT (*n* = 3) and KO (*n* = 3) naive H9 hES cells, obtained from RNA-seq data. Box plots represent the median (center), first and third quartiles (hinges) and ±1.5 IQR (whiskers). Unless otherwise specified, *P* values were calculated by a two-sided unpaired *t*-test. Levels of significance: NS (*P* ≥ 0.05), *P < 0.05, **P < 0.01, ***P < 0.001 and ****P < 0.0001. Source data

**Extended Data Fig. 2:**
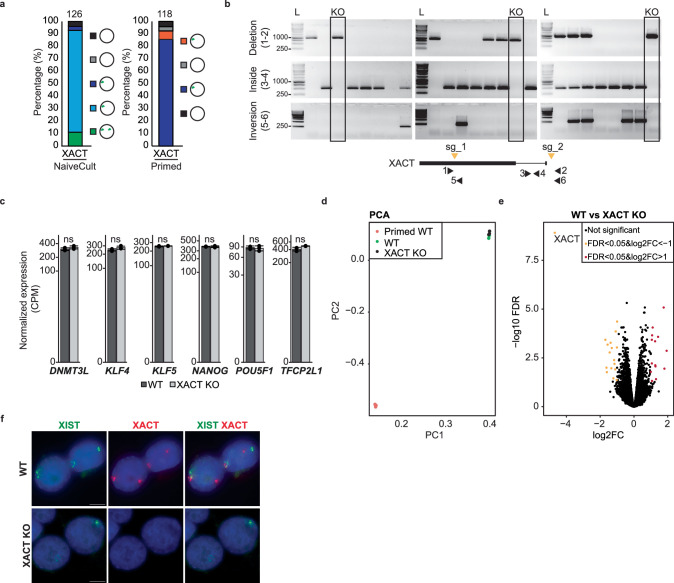
Characterization of XACT KO clones in naïve hESCs. **a**, Quantification of RNA-FISH data showing the percentage of cells with monoallelic or biallelic XIST (green) and/or X-linked gene (XACT; red) signals in naïve (NaiveCult) and primed H9 hESCs. **b**, Top: Electrophoretic gel pictures showing the PCR amplicons obtained with different screening primers on genomic DNA from XACT targeted primed H9 hESCs. Black rectangles represent 3 independent XACT KO clones which were used for subsequent experiments. Bottom: Schematic representation of XACT deletion strategy showing the positions of the CRISPR sgRNAs (yellow triangles) and screening primers used to characterize the clones (black arrows). **c**, Expression levels of core and naïve specific pluripotency factors in naïve H9 WT and XACT KO, from RNA-seq data (n = 3). Data are presented as mean values +/- SD of replicates. **d**, PCA displaying the first two principal components of the WT and XACT KO RNA-seq data in naïve and primed H9 hESCs. **e**, Volcano plot representing the significance versus Log2 fold change for all differentially expressed genes (n = 30, FDR < 0.05, log2FC > |1 | ) upon XACT deletion (n = 3). **f**, Representative images of XIST and XACT RNA-FISH in WT and XACT KO naïve (NaiveCult) H9 hESCs. Scale bar= 10 µm. Unless otherwise specified, p-values were calculated by two-sided unpaired *t*-test. Levels of significance: ns≥0.05; * < 0.05; ** < 0.01; *** < 0.001; **** < 0.0001. Source data

XISTʼs capacity to attenuate X chromosome transcription instead of triggering complete inactivation made us question whether XACT antagonizes XISTʼs silencing activity. We first validated that two XACT clouds were detected in most (97%) naive cells, as previously described in human preimplantation embryos^11^ (Fig. 2a,b and Extended Data Fig. 2a). Biallelic XACT expression in naive hES cells was not systematically correlated with increased XACT levels compared to primed hES cells (Fig. 2c).

We deleted a 90-kb region encompassing XACT transcription start sites (TSSs) in primed H9 cells (Fig. 2a,d and Extended Data Fig. 2b). Three independent homozygous XACT KO clones were successfully converted to a naive pluripotent state. Neither expression of naive-specific and core pluripotency factors nor cell morphology and growth were impacted by XACT deletion (Extended Data Fig. 2c). Furthermore, PCA revealed that XACT KO and WT naive H9 cells were grouped together and clustered away from primed hES cells, indicating that loss of XACT does not significantly alter the naive hES cell transcriptome (Extended Data Fig. 2d). This was confirmed through differential expression analysis, which revealed few genes with altered expression levels (14 upregulated and 16 downregulated; log_2_FC > |1| and FDR < 0.05) in XACT KO compared to WT naive H9 cells (Extended Data Fig. 2e and Supplementary Table 4). Gene Ontology analysis did not reveal any enriched pathways in the list of the 30 DEGs. This indicates that XACT is dispensable for primed to naive resetting and has no major role in naive hES cells.

XIST RNA levels were not altered in XACT KO clones (Fig. 2e). Similarly, the distribution of the XIST signal, which is more dispersed in naive hES cells compared to primed hES cells and fibroblasts^11,26^, was similar in XACT WT and KO clones (Fig. 2f and Extended Data Fig. 2f), indicating that XACT does not regulate XIST expression or localization in naive hES cells. As a proxy for global X chromosome expression, we measured the X:A ratio and observed no significant changes in naive XACT KO compared to WT hES cells (Fig. 2g). Furthermore, the percentage of biallelic X-linked SNPs detected by RNA-seq in XACT KO cells remained unchanged compared to WT, indicating that X chromosome silencing did not initiate in the absence of XACT in naive hES cells (Fig. 2h). These results indicate that XACT is not an antagonist of XIST and does not control X chromosome activity in naive hES cells.

### XIST accumulates on the dampened X chromosome in naive hES cells

**Fig. 3:**
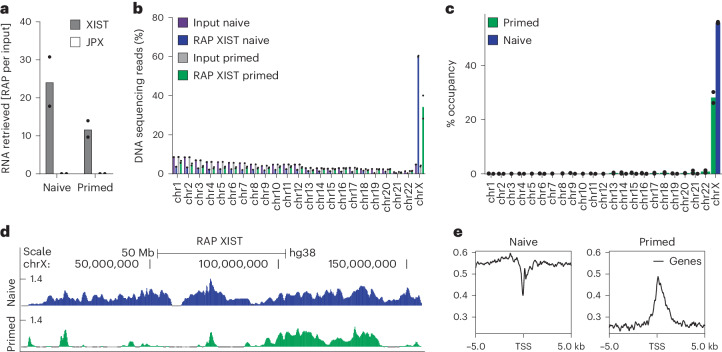
XIST is broadly distributed on the dampened X chromosome in naive hES cells. **a**, Analysis by RT–qPCR of XIST and JPX enrichment after XIST pulldown in naive and primed H9 hES cells. JPX was used as a negative control. Values were normalized to the input (*n* = 2). **b**, Percentage of aligned DNA sequencing reads per chromosome after XIST pulldown and in input material in naive and primed H9 hES cells (*n* = 2). **c**, Percentage of XIST RAP peak occupancy per chromosome for primed and naive H9 hES cells (*n* = 2). **d**, XIST RAP profiles (log_2_ enrichment over input) on the X chromosome in naive and primed H9 hES cells. **e**, Profile plots of XIST RAPseq reads over a 10-kb region flanking the TSSs of X-linked genes in naive and primed H9 hES cells. Source data

The XIST accumulation pattern in human preimplantation embryos and naive hES cells is thought to differ from that of primed hES cells, being more dispersed, as shown by RNA FISH^11,26^. To address whether the change in XIST repressive activity between naive and primed pluripotency could be linked to the redistribution of XIST RNA, we mapped at high resolution the genomic binding sites of XIST on dampened and inactive X chromosomes in naive and primed H9 hES cells, respectively, using RNA antisense purification followed by high-throughput sequencing (RAPseq). XIST was efficiently and specifically captured from the chromatin of both cell types (Fig. 3a).

**Extended Data Fig. 3:**
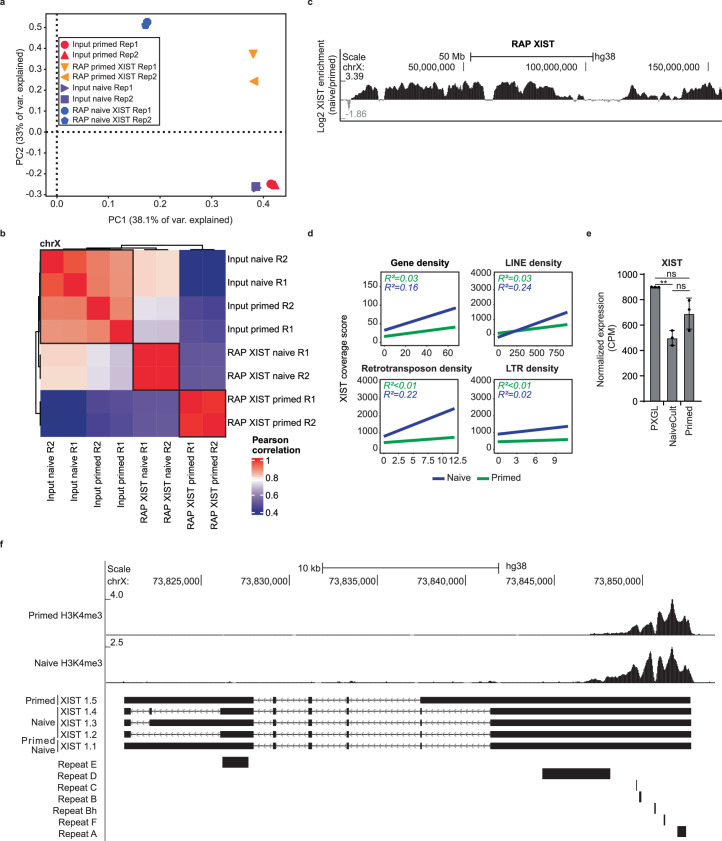
Characterization of XIST enrichment on the X chromosome in naïve and primed hESCs. **a**, PCA plot displaying the first two principal components of the XIST RAPseq data in naïve (PXGL) and primed H9 hESCs. **b**, Heatmap showing the Pearson correlation between naïve and primed XIST RAPseq replicates on the X chromosome. **c**, XIST RAPseq profiles showing the log2 enrichment over the X chromosome in naïve compared to primed H9 hESCs. **d**, Scatter plots showing the linear regression line and Pearson correlation for the comparison of XIST coverage scores with gene, LINE, retrotransposon, and LTR densities in naïve and primed H9 hESCs. **e**, Expression levels of XIST from naïve: PXGL (n = 3) and NaiveCult (n = 3), and primed (n = 3) H9 RNA-seq data. Data are presented as mean values +/- SD of replicates. **f**, CUT&RUN H3K4me3 profiles across the XIST gene in naïve and primed H9. XIST spliced isoforms detected in naïve and primed H9 along with the A-F repeats are shown. Arbitrary name of each isoform (XIST1.1, XIST1.2, etc) is indicated on the left, together with their expression pattern (Primed and/or Naïve). Unless otherwise specified, p-values were calculated by two-sided unpaired *t*-test. Levels of significance: ns≥0.05; * < 0.05; ** < 0.01; *** < 0.001; **** < 0.0001. Source data

The X chromosome showed the highest percentage of mapped DNA sequencing reads in both naive (60%) and primed (34%) hES cells, while the remaining reads were distributed across all autosomes (Fig. 3b). PCA and matrix correlation analysis showed that the naive RAP XIST replicates clustered together and away from the primed RAP XIST replicates. As the comparison was made in an isogenic framework, we can conclude that the variation in XIST distribution was because of the cellular context (Extended Data Fig. 3a,b). Indeed, XIST was broadly distributed on the X chromosome in naive hES cells, occupying >50% of the chromosome, while XIST contacted a reduced fraction (<30%) of the primed X chromosome, mostly on the long arm (Fig. 3c,d). Differential enrichment analysis confirmed that most of the X chromosome was enriched for XIST in naive compared to primed hES cells (Extended Data Fig. 3c). However, XIST was overall depleted from X-linked TSSs compared to flanking sequences in naive hES cells. This pattern was in sharp contrast to that of primed hES cells, where XIST accumulated over TSSs (Fig. 3e). These observations support a role for XIST distribution in the regulation of X-linked genes, where XIST RNA molecules relocate from X-linked promoters to flanking regions upon primed to naive transition.

We then probed the correlation of XIST distribution with several genomic features in both naive and primed hES cells. In naive hES cells, XIST coverage was moderately correlated with the density of transposable elements (TEs) such as LINEs (long interspersed nuclear elements) and retrotransposons, in contrast to primed hES cells (Extended Data Fig. 3d). This could reveal a role for these elements in XIST spreading upon initial XIST upregulation, as previously suggested^29,30^. The difference in XIST distribution was not linked to differential XIST expression levels (Extended Data Fig. 3e), suggesting that broader coverage of the X chromosome in naive hES cells was not because of an increased number of XIST RNA molecules. XIST transcript reconstruction revealed the presence of multiple XIST isoforms (Extended Data Fig. 3f). The XIST 1.1 isoform, with six exons, corresponded to the annotated XIST structure and was expressed in naive and primed hES cells. Other isoforms were specific to the primed or naive contexts and differed in their first and last exon. Importantly, all isoforms were produced from the same TSS and harbored all six repeat elements (A–F) important for XIST function and localization (Extended Data Fig. 3f)^31^. Altogether, these data reveal that XIST establishes distinct contacts on dampened and inactive X chromosomes, in line with the distinct activities of XIST in naive and primed pluripotent contexts.

### PRC histone marks accumulate on the dampened X chromosome

**Fig. 4:**
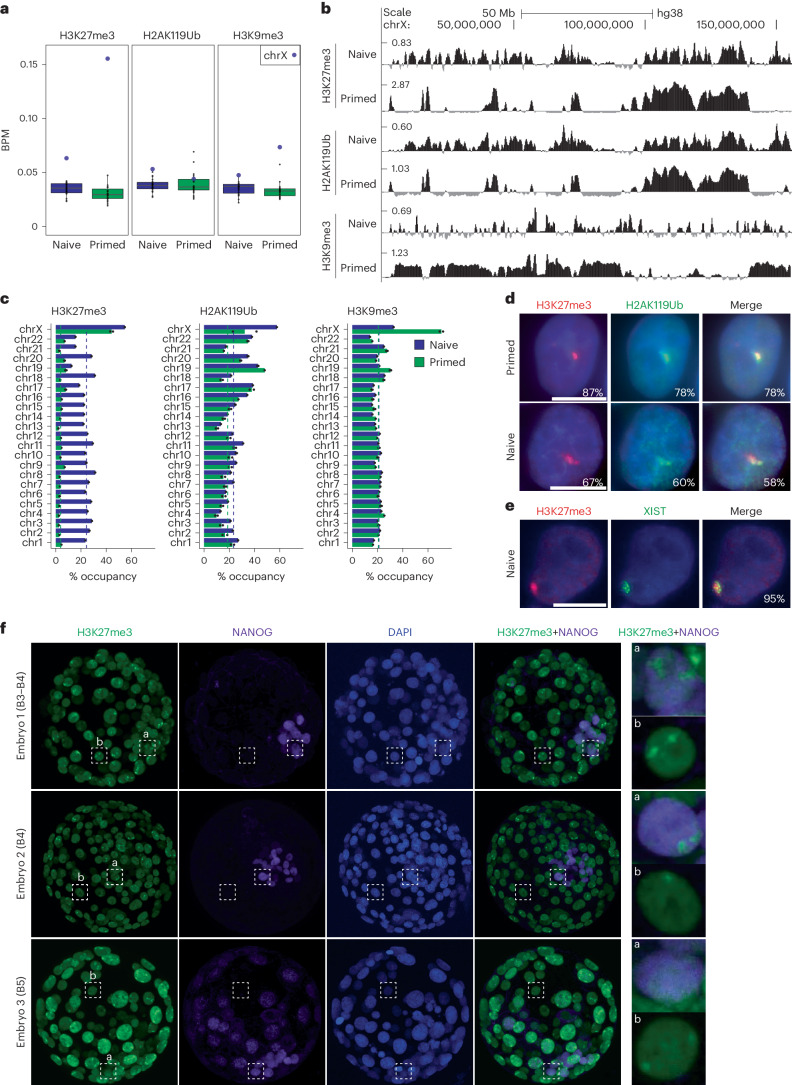
PRC-associated repressive histone modifications accumulate on dampened X chromosome in an XIST-dependent manner in naive hES cells. **a**, Mean enrichment of H3K27me3, H2AK119Ub and H3K9me3 per chromosome in naive and primed H9 hES cells (*n* = 2). All individual data points are shown. Box plots represent the median (center), first and third quartiles (hinges) and ±1.5 IQR (whiskers). BPM, bins per million mapped reads. **b**, CUT&RUN profiles (log_2_ enrichment over IgG) for H3K27me3, H2AK119Ub and H3K9me3 on the X chromosome in naive and primed H9 hES cells. **c**, Percentage of H3K27me3, H2AK119Ub and H3K9me3 peak occupancy per chromosome for primed and naive H9 hES cells. Black and green dotted lines represent the median genomic occupancy. **d**, Representative immunofluorescence images for H3K27me3 and H2AK119Ub in primed and naive H9 hES cells. Percentages of cells displaying the representative pattern are indicated. Scale bar = 10 µm. **e**, Representative immunofluorescence images for H3K27me3 coupled with XIST RNA FISH in naive H9 hES cells. Percentages of cells displaying the representative pattern are indicated. Scale bar = 10 µm. **f**, Immunofluorescence images for H3K27me3 and NANOG in human preimplantation blastocysts (stages B3 to B5). Source data

Because XIST is known to indirectly recruit PRC complexes and given its ability to control X chromosome activity in naive hES cells, we interrogated the chromatin landscape of dampened and inactive X chromosomes using CUT&RUN (cleavage under targets and release using nuclease). Levels of H3K27me3, H2AK119Ub and, to a lesser extent, H3K9me3 were higher on the X chromosomes than on autosomes in naive hES cells (Fig. 4a), even though this tendency was less pronounced than in primed hES cells, at least for H3K27me3 and H3K9me3.

**Extended Data Fig. 4:**
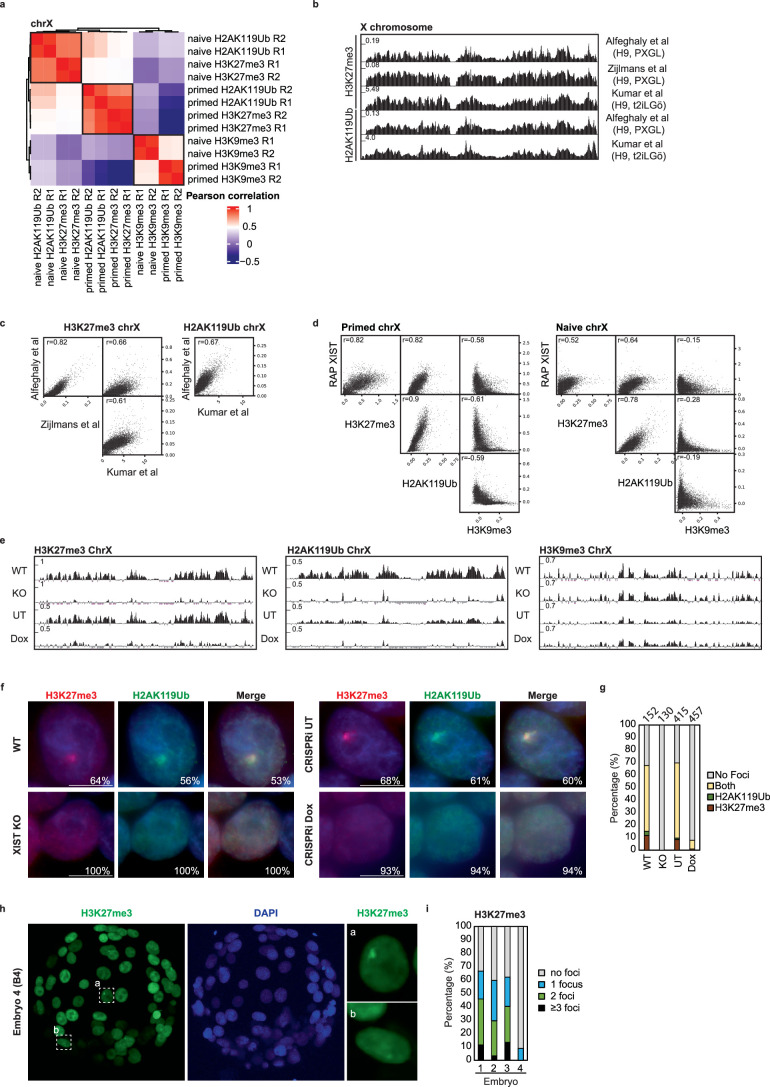
Characterization of the X chromatin landscape in WT, XIST KO and XIST CRISPRi UT and Dox-treated clones in naïve hESCs. **a**, Heatmap showing the Pearson correlation between X chromosome H3K27me3, H2AK119Ub, and H3K9me3 CUT&RUN replicates from naïve and primed H9. **b**, X chromosome H3K27me3 and H2AK119Ub profiles from H9 naïve cells reprogrammed using PXGL^62^ or t2iLGO^63^. **c**, Scatter plots showing the Pearson correlation, for the X chromosome, between our CUT&RUN dataset and the other datasets. **d**, Scatter plots showing the Pearson correlation between RAP XIST and repressive histone modifications (H3K27me3, H2AK119Ub, H3K9me3) on the X chromosome in naïve and primed H9. **e**, CUT&RUN profiles (log2 enrichment over IgG) for H3K27me3, H2AK119Ub and H3K9me3 on the X chromosome in naïve WT, XIST KO and XIST CRISPRi H9 cells. **f**, Representative immunofluorescence images for H3K27me3 and H2AK119Ub in naïve H9 WT, XIST KO, XIST CRISPRi UT or treated with Dox. Percentages of cells with the representative pattern are indicated. Scale bar= 10 µm. **g**, Quantification of immunofluorescence data showing the percentage of cells with H3K27me3 and H2AK119Ub foci in XIST KO and XIST CRISPRi naïve hESCs. **h**, H3K27me3 immunofluorescence of one human early blastocyst (B4). **i**, Quantification of immunofluorescence data from human embryos showing the percentage of cells with 0, 1, 2, or ≥3 H3K27me3 foci. Source data

Coverage profiles showed a distinct distribution of H3K27me3, H2AK119Ub and H3K9me3 on the X chromosome in naive compared to primed hES cells (Fig. 4b). Notably, H3K27me3 and H2AK119Ub covered around 60% of the X chromosome in naive hES cells and 40–50% in primed hES cells, while most of the X chromosome (>60%) was covered by H3K9me3 in primed hES cells versus 33% in naive hES cells (Fig. 4c). H3K27me3 and H2AK119Ub patterns were highly correlated together and anticorrelated with H3K9me3 in both primed and naive hES cells (Extended Data Fig. 4a). H3K27me3 and H2AK119Ub signals accumulated into several smaller domains that spanned most of the XIST-coated dampened X chromosome in naive hES cells (Fig. 4b). We observed similar profiles of H3K27me3 and H2AK119Ub using other published datasets of naive H9 hES cells (Extended Data Fig. 4b,c). In contrast, H3K27me3 and H2AK119Ub accumulated over several large domains of the Xi, with the major one spanning around 50 Mb in the middle of the long arm, while the H3K9me3 signal spanned most of the short arm, as well as the most proximal and distal parts of the long arm (Fig. 4b). This is reminiscent of what was described in previous studies^32,33^. Altogether, this showed that X chromosomes of naive hES cells are enriched for repressive histone modifications, which are massively redistributed along the X chromosome upon transition from primed to naive hES cells.

Patterns of H3K27me3 and H2AK119Ub in naive hES cells were highly correlated to that of XIST in naive as in primed hES cells, suggesting that the deposition of PRC-associated histone modifications on the X chromosome is mediated by XIST in both contexts (Extended Data Fig. 4d). We confirmed this hypothesis by showing a global loss of H3K27me3 and H2AK119Ub on the X chromosome in XIST KO and Dox-treated CRISPRi clones compared to their WT counterpart (Extended Data Fig. 4e), while H3K9me3 distribution and levels remained unchanged.

Immunofluorescence approaches confirmed the CUT&RUN data (Fig. 4d) as one focus of H3K27me3 and H2AK119Ub was observed in >60% of naive H9 hES cells, with frequent co-occurrence of the two marks (Fig. 4d). This H3K27me3 focal accumulation corresponded to the XIST-coated dampened X chromosome, as shown by immuno-RNA FISH (Fig. 4e). In the absence of XIST, H3K27me3 and H2AK119Ub coaccumulation was lost on the X chromosome in more than 90% of cells (Extended Data Fig. 4f,g).

Because conflicting results existed regarding the accumulation of PRC-mediated repressive histone modifications on the X chromosomes before XCI establishment^10,11,26,34^, we re-examined the accumulation of H3K27me3 in human preimplantation embryos. In four blastocyst-stage embryos, we could observe cells with one or two H3K27me3 foci, in both NANOG^+^ and NANOG^−^ cells (Fig. 4f and Extended Data Fig. 4h,i). The presence of two foci in a fraction of NANOG^+^ cells in three of four embryos strongly suggested that active X chromosomes were indeed enriched for H3K27me3 in a fraction of epiblast cells of female blastocysts. Embryo 4, in which only one H3K27me3 focal enrichment was detected in around 10% of cells, was likely male (Extended Data Fig. 4h,i). Overall, these data indicate that PRC-mediated repressive histone modifications are enriched on and broadly cover XIST-associated X chromosomes in most cells of female preimplantation embryos and naive hES cells.

### Highly expressed genes escape XIST-mediated dampening

**Fig. 5:**
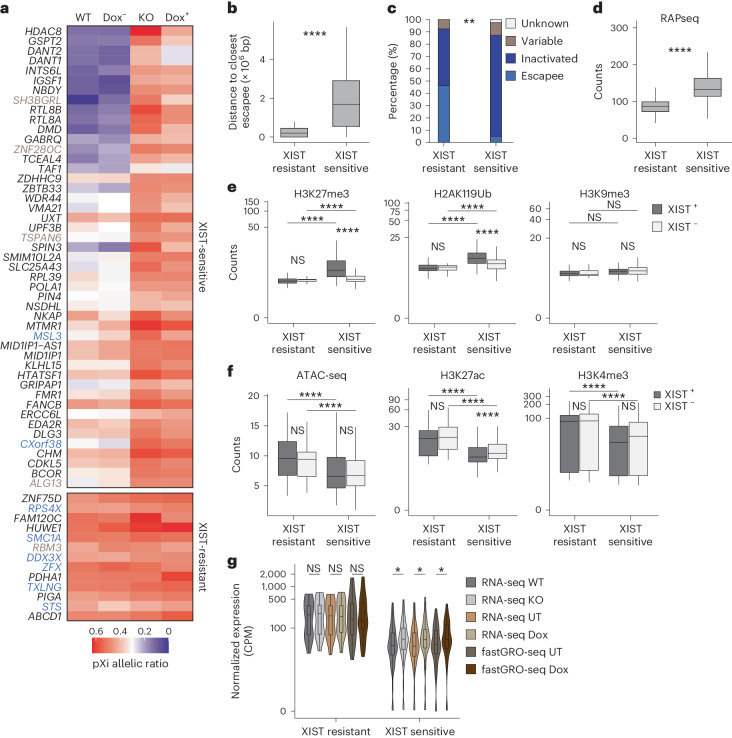
Highly expressed genes escape XIST-mediated dampening. **a**, Heat map showing the pXi allelic ratio of XIST-sensitive and XIST-resistant genes in XIST WT, KO, UT CRISPRi (Dox^−^) and Dox-treated CRISPRi (Dox^+^) naive H9 cells. Constitutive and variable escapees from the Tukiainen study^43^ are highlighted in light blue and brown, respectively. **b**, Distance to closest escapee of XIST-sensitive (*n* = 47) and XIST-resistant (*n* = 13) genes. **c**, Percentage of escapees (constitutive and variable) and inactivated X-linked genes from the Tukiainen study^43^ among XIST-sensitive and XIST-resistant genes; chi-square test *P* value < 0.01 (**). **d**, XIST RAPseq counts on TSS ± 5 kb of XIST-sensitive (*n* = 47) and XIST-resistant (*n* = 13) genes in naive H9 cells. **e**, H3K27me3, H2AK119Ub and H3K9me3 counts on TSS ± 5 kb of XIST-sensitive (*n* = 47) and XIST-resistant (*n* = 13) genes in the presence (XIST WT and UT CRISPRi) or absence (XIST KO and Dox-treated CRISPRi) of XIST in naive H9 hES cells. **f**, ATAC-seq, H3K27ac and H3K4me3 counts on TSS ± 5 kb of XIST-sensitive (*n* = 47) and XIST-resistant (*n* = 13) genes in the presence (XIST WT and UT CRISPRi) or absence (XIST KO and Dox-treated CRISPRi) of XIST in naive H9 hES cells. **g**, Violin plots showing the log CPM expression level of XIST-sensitive (*n* = 47) and XIST-resistant (*n* = 13) genes in XIST WT, KO, UT CRISPRi and Dox-treated CRISPRi naive H9 cells, obtained from RNA-seq and fastGRO-seq data. All box plots represent the median (center), first and third quartiles (hinges) and ±1.5 IQR (whiskers). Unless otherwise specified, *P* values were calculated by a two-sided unpaired *t*-test. Levels of significance: NS (*P* ≥ 0.05), *P < 0.05, **P < 0.01, ***P < 0.001 and ****P < 0.0001. Source data

The broad distribution of XIST and repressive histone modifications along the dampened X chromosome led us to explore whether all X-linked genes are equally responsive to XIST-mediated regulation in naive hES cells, according to their allelic expression profiles. Most of the X-linked genes that could be analyzed using SNPs were ‘XIST-sensitive’ genes (47 of 60; 78%), in agreement with XIST covering most of the X chromosome in naive hES cells. These genes displayed lower expression from the XIST-associated X chromosome and became equally expressed from both X chromosomes in the absence of XIST (Fig. 5a and Supplementary Table 5). In addition, we could identify a fraction of ‘XIST-resistant’ genes (13 of 60; 22%), with a pXi allelic ratio of ~0.5 in the presence or absence of XIST, indicating equal expression from the dampened (pXi) and active (pXa) X chromosomes in naive hES cells (Fig. 5a and Supplementary Table 5).

**Extended Data Fig. 5:**
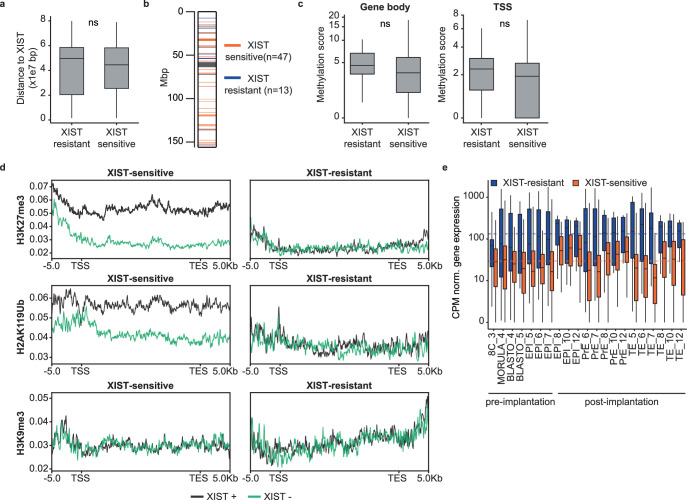
Genetic and epigenetic features of XIST-sensitive and XIST-resistant genes. **a**, Distance to XIST locus of XIST-sensitive (n = 47) and resistant (n = 13) genes. **b**, Chromosomal map indicating the positions of XIST-sensitive and resistant genes on the X chromosome. The centromere is indicated by a grey rectangle. **c**, Comparison of MeD-seq methylation scores in TSS (right) and gene body (left) between XIST-sensitive (n = 47) and resistant (n = 13) genes. **d**, Profile plots of H3K27me3, H2AK119Ub, and H3K9me3 over XIST-sensitive and resistant genes in XIST positive (WT, XIST CRISPRi UT) and XIST negative (XIST KO, Dox-treated XIST CRISPRi) naïve H9 cells. **e**, Expression levels of XIST-resistant (n = 13) and sensitive (n = 47) genes in human pre-implantation and post-implantation embryos, based from scRNA-seq^20,25^. Purple and orange dotted lines indicate the median expression of XIST-resistant and XIST-sensitive genes, respectively. BLASTO: blastocyst; EPI: epiblast; PrE: primitive endoderm; TE: trophectoderm. Numbers correspond to the embryonic days. All boxplots represent the median (center) first and third quartile (hinges) and ±1.5 × IQR (whiskers). Unless otherwise specified, p-values were calculated by two-sided unpaired *t*-test. Levels of significance: ns≥0.05; * < 0.05; ** < 0.01; *** < 0.001; **** < 0.0001. Source data

While no significant difference was observed in the distance to the XIST locus between XIST-resistant and XIST-sensitive genes (Extended Data Fig. 5a), XIST-resistant genes tended to be located in the vicinity of XCI escapees and were enriched on the short arm, while XIST-sensitive genes appeared to be scattered along the X chromosome (Fig. 5b and Extended Data Fig. 5b). Of note, the majority of XIST-resistant genes in naive H9 cells are known constitutive or variable escapees (Fig. 5c), suggesting that this set of genes is refractory to XISTʼs repressive activities (dampening or inactivation), whatever the context.

XIST-sensitive genes were significantly more contacted by XIST RNA and had a higher accumulation of H3K27me3 and H2AK119Ub compared to XIST-resistant genes, while H3K9me3 and DNA methylation levels were similar in both categories (Fig. 5d,e and Extended Data Fig. 5c). Accordingly, only XIST-sensitive genes displayed reduced H3K27me3 and H2AK119Ub levels in the absence of XIST (XIST KO and Dox-treated CRISPRi), with levels reaching that of XIST-resistant genes, while H3K9me3 remained constant across gene categories and genotypes (Fig. 5e and Extended Data Fig. 5d).

Conversely, the TSSs of XIST-sensitive genes were less accessible than those of XIST-resistant genes and were marked by lower levels of active histone modifications (H3K27ac and H3K4me3; Fig. 5f). H3K27ac but not H3K4me3 levels were elevated specifically at XIST-sensitive genes upon loss of XIST but remained lower compared to XIST-resistant genes. This indicates that, while XIST is responsible for the deposition of repressive histone marks at target genes, it only mildly prevents the acquisition of active promoters’ features. In agreement with these observations, XIST-sensitive genes were expressed at lower levels than XIST-resistant genes in naive hES cells and did not reach the expression levels of XIST-resistant genes in the absence of XIST (Fig. 5g). Interestingly, a similar tendency was observed in preimplantation and postimplantation human embryos (Extended Data Fig. 5e). Altogether, these results show that, in naive hES cells and possibly in preimplantation embryos, XIST attenuates the expression of the majority of X-linked genes, with highly expressed genes resisting XIST targeting and dampening activity.

### SPEN is involved in XIST-mediated dampening

**Fig. 6:**
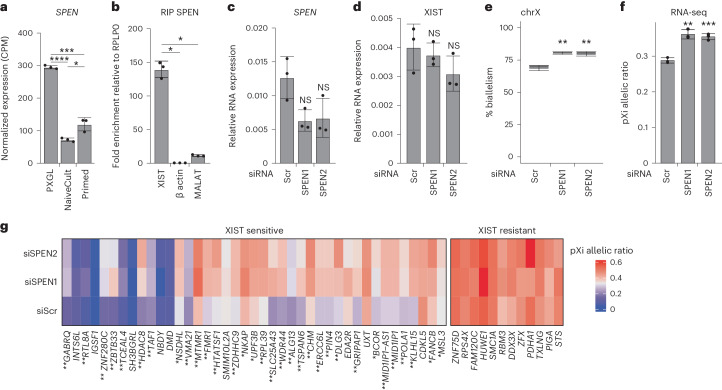
SPEN is involved in XIST-mediated dampening. **a**, Expression level of SPEN in naive (PXGL and NaiveCult) and primed H9 cells obtained from RNA-seq data (*n* = 3). Data are presented as the mean values ± s.d. of replicates; Wilcoxon *P* value ≥ 0.05 (NS). **b**, RT–qPCR showing fold enrichment levels of XIST, β-actin and MALAT1 normalized to *RPLP0* following RIP of SPEN (*n* = 3). Data are presented as the mean values ± s.d. of replicates; Wilcoxon *P* value < 0.05 (*). **c**, RT–qPCR analysis of SPEN expression after siRNA KD using two different mixes of siRNAs (SPEN1 and SPEN2; *n* = 3). Values are normalized to β-actin. Data are presented as the mean values ± s.d. of replicates; Wilcoxon *P* value ≥ 0.05 (NS). **d**, RT–qPCR analysis of XIST expression after SPEN KD (*n* = 3). Values are normalized to β-actin. Data are presented as the mean values ± s.d. of replicates; Wilcoxon *P* value ≥ 0.05 (NS). **e**, Percentage of biallelically expressed SNPs from X chromosome in scramble (*n* = 3) and SPEN (*n* = 3) KD naive H9 cells, obtained from RNA-seq data. Box plots represent the median (center), first and third quartiles (hinges) and ±1.5 IQR (whiskers). **f**, pXi allelic ratio from RNA-seq data of scramble and SPEN KD naive H9 hES cells (*n* = 3). Data are presented as the mean values ± s.d. of replicates. **g**, Heatmap showing the pXi allelic ratio of XIST-sensitive and XIST-resistant gene expression upon SPEN KD in naive H9 hES cells. An increase in pXi allelic ratio (FC > 1.2) from one (*) or both (**) siSPEN mixes is indicated. Unless otherwise specified, *P* values were calculated by a two-sided unpaired *t*-test. Levels of significance: NS (*P* ≥ 0.05), *P < 0.05, **P < 0.01, ***P < 0.001 and ****P < 0.0001. Source data

**Extended Data Fig. 6:**
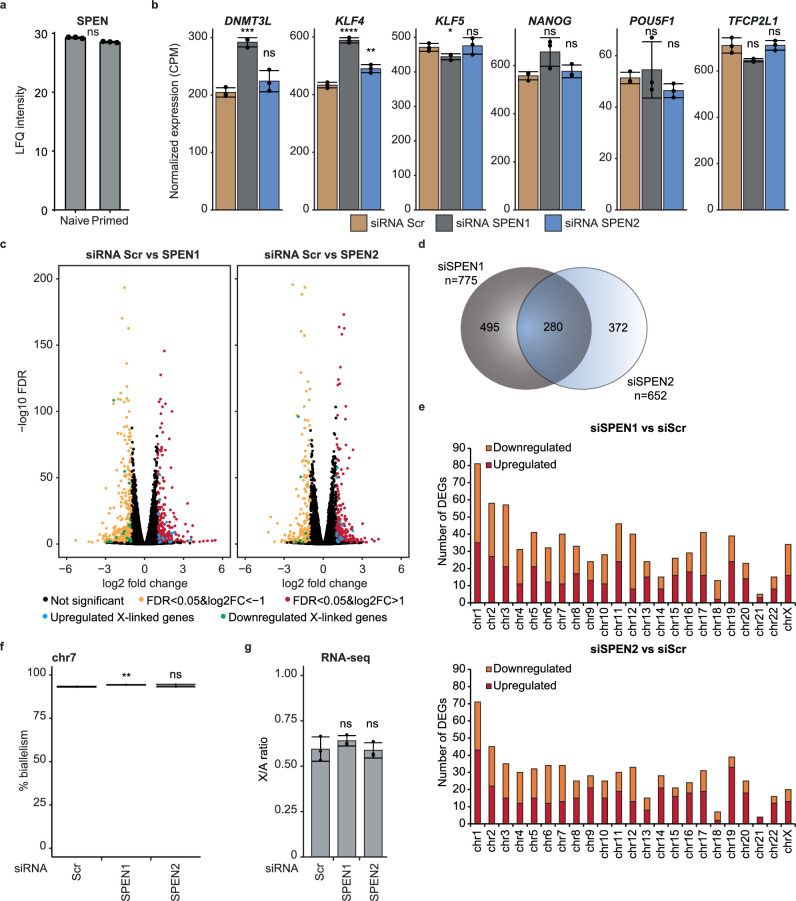
Characterization of SPEN expression and KD. **a**, Protein abundance level of SPEN from publicly available proteomics data^62^ in naïve (n = 3) and primed (eroded) (n = 3) H9 cells. Data are presented as mean values +/- SD of replicates. Wilcoxon p-value = ns≥0.05. **b**, Expression level in CPM of naïve and core pluripotency factors in control (siScr) and SPEN knockdown naïve H9 (n = 3). Data are presented as mean values +/- SD of replicates. **c**, Volcano plots representing the significance versus Log2 fold change for all differentially expressed genes (FDR < 0.05, log2FC > |1 | ) upon SPEN KD (n = 3). Upregulated and downregulated X-linked genes are highlighted. **d**, Venn diagram showing the intersection of differentially expressed genes between siSPEN1 and siSPEN2 datasets. **e**, Bar plot showing the number of differentially expressed genes on all chromosomes in siSPEN1 and siSPEN2 conditions compared to siScr. **f**, Percentage of biallelically expressed SNPs from chromosome 7 in siScr (n = 3) and siSPEN (n = 3) naïve H9, obtained from RNA-seq data. **g**, X/A ratio from RNA-seq data of siRNA SPEN KD in naïve H9 cells (n = 3). Data are presented as mean values +/- SD of replicates. Unless otherwise specified, p-values were calculated by two-sided unpaired *t*-test. Levels of significance: ns≥0.05; * < 0.05; ** < 0.01; *** < 0.001; **** < 0.0001. Source data

Previous studies showed that SPEN interacts with the A repeat of mouse Xist RNA and is rapidly recruited to the X chromosome at the initiation of XCI to promote histone deacetylation^12–14^. Whether SPEN interacts with human XIST and is recruited on the dampened X chromosome in naive hES cells is unknown. We first analyzed SPEN expression and protein abundance by RNA-seq and mass spectrometry, respectively. SPEN mRNA levels were significantly higher in PXGL-reprogrammed compared to NaiveCult-reprogrammed or primed H9 cells (Fig. 6a), whereas SPEN protein abundance appeared largely similar between PXGL-reprogrammed and primed H9 cells (Extended Data Fig. 6a). RNA immunoprecipitation (RIP) experiments using SPEN antibody led to a 140-fold enrichment for XIST RNA relative to control *RPLP0* RNA. In contrast, β-actin mRNAs or MALAT1 (metastasis-associated lung adenocarcinoma transcript 1) noncoding RNAs that were not reported to interact with SPEN were not significantly immunoprecipitated (Fig. 6b). This supports SPENʼs association to XIST in naive hES cells.

We then transiently knocked down SPEN in naive H9 hES cells using two different mixes of small interfering RNAs (siRNAs; SPEN1 and SPEN2) (Fig. 6c). SPEN knockdown (KD) had no major impact on pluripotency factor or XIST expression level (Extended Data Fig. 6b and Fig. 6d). Differential expression analysis revealed that 775 genes (351 upregulated and 424 downregulated; log_2_FC > |1| and FDR < 0.05) or 652 genes (379 upregulated and 273 downregulated; log_2_FC > |1| and FDR < 0.05) showed altered expression levels upon siSPEN1 or siSPEN2 KD compared to control siScr (Extended Data Fig. 6c,d and Supplementary Tables 6 and 7). DEGs were distributed on all chromosomes and not specifically enriched on the X chromosome, consistent with the role of SPEN as a regulator of different key pathways^35–37^ (Extended Data Fig. 6e).

To evaluate the role of SPEN in the regulation of X chromosome activity, we analyzed the percentage of biallelically detected X-linked SNPs in SPEN KD naive hES cells compared to control cells by RNA-seq. We observed a slight increase in biallelically expressed SNPs upon SPEN KD, suggesting that SPEN loss led to higher expression of a subset of X-linked genes, presumably from the XIST-coated X chromosome (Fig. 6e and Extended Data Fig. 6f). This was confirmed by calculating the pXi allelic ratio, which was significantly increased in the SPEN KD condition compared to siScr (Fig. 6f), albeit to a lesser extent than that in XIST-null conditions (Fig. 1i). This may have been because of incomplete SPEN KD or the involvement of other factors in dampening, which likely explains why the X:A ratio was not affected upon SPEN KD (Extended Data Fig. 6g).

Lastly, we explored the dependency of XIST-sensitive and XIST-resistant genes on SPEN activity. Interestingly, 32 of 41 detected XIST-sensitive genes (78%) displayed a higher pXi allelic ratio (FC > 1.2) in one or both siSPEN mixes compared to siScr control (Fig. 6g and Supplementary Table 8). In contrast, no change in pXi allelic ratio was observed for XIST-resistant genes in the absence of SPEN, consistent with XIST recruiting SPEN to X-linked genes to exert its repressive activity (Fig. 6g). Overall, these results reveal the implication of SPEN in mediating the dampening activity of XIST in naive hES cells.

## Discussion

The intriguing uncoupling between XIST accumulation and XCI during early human development raises several fundamental questions that we aimed to address: Are there mechanisms protecting the X chromosome from being silenced? Is XIST functional? Is XIST involved in alternative dosage compensation mechanisms? If so, what are the underlying mechanisms?

We previously hypothesized that the lncRNA XACT could be an antagonist of XIST during preimplantation development^11^. In that case, loss of XACT expression in naive hES cells should lead to inactivation of XIST-coated X chromosomes. Deleting a large genomic fragment containing all XACT TSSs revealed that XACT does not regulate X chromosome activity or significantly impact gene expression in naive hES cells. However, this does not exclude the possibility of XACT being functional in other cellular contexts. A previous study showed that insertion of an XACT transgene in mouse cells influenced XIST accumulation in cis^11^, which suggests a potential function of XACT during the initiation phase of XCI by dictating the choice of the X chromosome to be inactivated. Moreover, XACT deletion in primed hES cells affects neural differentiation through mechanisms that remain to be determined^38^. Lastly, XACT is highly expressed in primordial germ cells^39^. This could reflect either a cellular context resembling naive pluripotency, permissive for XACT expression, or a more specific role for XACT during germ line specification.

An alternative scenario to explain why XACT KO does not result in XCI in naive hES cells invokes the production of a nonfunctional form of XIST at this stage. We could discard this hypothesis by demonstrating that XIST triggers deposition of PRC-mediated histone modifications, interacts with SPEN and modulates the activity of the coated X chromosome. Indeed, we revealed an enrichment in H3K27me3 and H2AK119ub on active X chromosomes in naive hES cells and in human preimplantation blastocysts. This contrasts with a previous report where active X chromosomes were found, by immunofluorescence, devoid of H3K27me3 in human embryos^10^. Such a discrepancy may have resulted from different embryo handling and/or hybridization conditions. Alternatively, the enrichment of active X chromosomes in PRC-mediated histone modifications may be transient or heterogeneous and, thus, variably captured, in agreement with foci of H3K27me3 being visible only in a subset of blastocyst nuclei within a given embryo. These observations are nevertheless in agreement with recent findings in cynomolgus monkey preimplantation embryos, in which H3K27me3 marked both active X chromosomes^9^. In that study, Okamoto and colleagues also unraveled the dynamics of XCI, with earlier establishment in the trophectoderm compared to embryonic lineages^9^. Interestingly, we observed a non-negligible number of NANOG^−^ cells, which correspond to trophectoderm cells, harboring one focus of H3K27me3, which could indicate that XCI is initiated before implantation in human embryos, as in cynomolgus monkey embryos.

Even though active and inactive X chromosomes featured enrichment in PRC-repressive marks, our CUT&RUN data revealed a redistribution of the X chromosome repressive chromatin landscape upon transition from primed to naive hES cells. Because we compared active and inactive X chromosome features in an isogenic context, we could confidently link the observed differences to the cellular state. Repressive modifications were broadly distributed on active X chromosome in naive cells but their enrichment was higher on the inactive X chromosome of primed hES cells. We demonstrated that XIST is responsible for the deposition of these modifications in naive hES cells on the basis of the following observations: (1) exclusive enrichment on XIST-coated X chromosome; (ii) H3K27me3 and H2AK119Ub patterns on active X chromosome following that of XIST; and (iii) XIST loss leading to depletion of H3K27me3 and H2AK119Ub specifically on the X chromosome.

The redistribution of XIST on the active X chromosome upon hES cell resetting, which led to a large proportion of the naive active X chromosome being contacted by XIST, is consistent with previous findings of XIST clouds being more dispersed in naive hES cells and human preimplantation embryos, as observed in RNA FISH experiments^11,26^. It is still unknown how XIST propagates on the X chromosome and what drives such distinct XIST distribution in hES cells. It was proposed that TEs, particularly LINEs, might serve as way stations for XIST propagation on the X chromosome^29,30^. Here, we observed a slight correlation between XIST coverage and the density of LINEs and retrotransposons in naive hES cells. Thus, upon zygotic genome activation, the newly produced XIST RNA molecules might be preferentially targeted to TE-enriched regions. Spatial organization of the X chromosome may also orchestrate XIST distribution, as previously shown in mouse^40^.

XISTʼs broad distribution has consequences for X chromosome activity in naive hES cells. Indeed, we showed that XIST is responsible for attenuating the expression level of most X-linked genes it contacts. Genes that were less contacted by XIST escaped dampening and were expressed at higher levels compared to XIST-sensitive genes not only in naive hES cells but also in embryos. Interestingly, genes escaping XCI were also expressed at higher levels than other X-linked genes, which was proposed to be connected to the strong purifying selection characterizing escapees^41^. The fact that dampening-resistant genes also escaped XCI or were located in proximity to XCI escapees suggests common mechanisms for resisting XISTʼs repressive activities. Reversely, our data indicated that dampening is mediated at the level of transcription initiation, involves the XIST interactor SPEN and is associated with XIST-mediated deposition of PRC modifications, suggesting a conserved mode of action for XIST in both contexts.

The question remains as to what is preventing full silencing of X chromosomes at this stage. Multiple nonexclusive scenarios could be envisioned, such as the absence and/or defects (in expression or post-translational modifications) of certain XIST effectors. Another possible explanation is the presence or absence of post-transcriptional modifications on XIST RNA that can alter its interaction with protein partners or disrupt its folding. Lastly, other unknown factors might prevent XIST from silencing the X chromosome. Whatever the mechanisms, signals that trigger the switch in XIST activity must be linked to developmental progression. This is reminiscent of mouse ES cells, where inducible Xist induces XCI but not to its completion, with differentiation being required for full XCI in a manner that involves SmcHD1 (ref. ^19^).

Lastly, one might question the functional relevance of dampening. We could not observe any change in cell morphology, proliferation or transcriptomic signature when XIST was deleted or repressed in naive hES cells, suggesting that XCD is not required for stem cell fitness in vitro. Whether it is necessary for the progression of peri-implantation development or whether altered dampening could leave scars and affect future development is unknown. ‘Premarking’ the X chromosome with PRC-repressive modifications and attenuating X chromosome activity could facilitate the initiation of XCI, providing that asymmetry between the two X chromosomes is subsequently installed. It is yet unclear whether dampening is a hominoid-specific mechanism or is deployed in all primates. A recent investigation in cynomolgus monkey preimplantation embryos revealed a transient dampened-like status; however, this did not lead to complete X chromosome dosage compensation, as X-linked gene expression levels remained higher in female compared to male individuals^9^. Of note, XCD is the dosage compensation strategy at stake in *Caenorhabditis elegans*, where it is achieved by the dosage compensation complex (DCC)^42^. Overall, this highlight both diversity and convergence in the evolution of mechanisms and actors underlying X chromosome dosage compensation across species and clades, with dampening being triggered by unrelated sets of factors in nematodes and mammals, while XIST ribonucleoprotein complexes display distinct activities according to the cellular context in a given species.

## Methods

### Human cell lines and culture conditions

Experiments were carried out using female H9 hES cells obtained from the WiCell Research Institute. Research on hES cells was approved by the Agence de la Biomédecine and informed consent was obtained from all subjects.

Primed H9 hES cells were cultured on Matrigel-coated culture dishes in mTeSR1 medium (StemCell Technologies) according to the manufacturer’s instructions in 5% O_2_ and 5% CO_2_ at 37 °C. They were routinely passaged in clumps using gentle cell dissociation reagent (StemCell Technologies) according to the manufacturer’s instructions. For experiments requiring a single-cell suspension, cells were incubated with Accutase (StemCell Technologies) and plated in fresh mTeSR1 medium supplemented with 10 μM Y-27632 (StemCell Technologies).

Naive H9 hES cells were generated by chemical resetting of the H9 primed hES cells using the NaiveCult (StemCell Technologies) or PXGL protocol, as previously described, and cultured on inactivated mouse embryonic fibroblasts (MEFs)^23,24^. PXGL naive hES cells were cultured in PXGL medium consisting of a 1:1 mixture of DMEM/F12 (Sigma-Aldrich) and Neurobasal medium supplemented with 0.5% N-2 supplement, 1% B-27 supplement, 2 mM l-glutamine, 100 µM β-mercaptoethanol and 1× penicillin–streptomycin (all from Gibco, Thermo Fisher Scientific), as well as 1 μM PD0325901 (Axon Medchem, 1408; CAS: 391210-10-9), 2 μM XAV939 (Cell Guidance Systems, SM38-10; CAS: 284028-89-3), 2 μM Gö6983 (Tocris, 2285; CAS: 133053-19-7), 10 ng ml^−1^ human leukemia inhibitory factor (LIF; Peprotech, 300-05). Naive hES cells were routinely passaged as single cells every 3 days at a ratio of 1:3 using 1× TrypLE Express (Gibco, Thermo Fisher Scientific) and plated in fresh medium supplemented with 10 μM Y-27632 (StemCell Technologies).

### Human preimplantation embryos

The use of human embryos donated to research as surplus of in vitro fertilization (IVF) treatment was allowed by the French embryo research oversight committee (Agence de la Biomédecine) under approval number RE18-010R. Embryos used were initially created in the context of an assisted reproductive cycle with a clear reproductive aim and then voluntarily donated for research once the patients had fulfilled their reproductive needs or tested positive for the presence of monogenic diseases. Informed written consent was obtained from both parents of all couples that donated spare embryos following IVF treatment. French legislation does not include the research project in the consent form; the embryos are donated to research, in general. The Agence de la Biomédecine oversight committee rules which project can use which embryos. Donor compensation is forbidden under French law.

All human preimplantation embryos used in this study were obtained from and cultured in the Assisted Reproductive Technology unit of the University Hospital of Nantes, France, which is authorized to collect embryos for research under approval number AG110126AMP of the Agence de la Biomédecine. Molecular analysis of the embryos was performed in compliance with the embryo research oversight committee and the International Society for Stem Cell Research (ISSCR) guidelines^44^.

Human embryos were thawed following the manufacturer’s instructions (Sydney IVF Thawing Kit for slow freezing, from Cook Medical; RapidWarmCleave or RapidWarmBlast for vitrification, from Vitrolife). Human embryos frozen at the eight-cell stage were loaded in a 12-well dish (Embryoslide, from Vitrolife) with nonsequential culture medium (G-TL, from Vitrolife) under mineral oil (liquid paraffin, from Origio), at 37 °C in 5% O_2_ and 6% CO_2_.

### Generation of XACT and XIST KO cell line

Single-guide RNA (sgRNA) sequences flanking the XACT and XIST promoters and first exon were obtained using the web-based tool CRISPOR (http://crispor.tefor.net/) and are provided in Supplementary Table 9. sgRNAs were cloned under a U6 promoter into the pSpCas9(BB)-2A-GFP (green fluorescent protein) (a gift from Feng Zhang; Addgene 48138) and the pSpCas9(BB)-2A-mCherry (generated in house, by replacing GFP with an mCherry reporter using the NEBuilding HiFi DNA Assembly Cloning Kit (New England Biolabs)^45^. Using the Amaxa 4D-Nucleofector system (Lonza), one million primed H9 hES cells were transfected with 2.5 µg of each plasmid (to a total of 5 µg). Cells were sorted by fluorescence-activated cell sorting (INFLUX 500-BD, BioSciences) 48 h after transfection. Double-positive cells were plated onto a Matrigel-coated 6-cm Petri dish in mTeSR medium supplemented with 1× CloneR (StemCell Technologies). Individual colonies were manually placed into 96-well plates ~10 days after transfection. Deletions and inversion events were screened by PCR. Primer sequences can be found in Supplementary Table 10. XACT and XIST KO H9 primed hES cells were reprogrammed to naive using the NaiveCult and PXGL protocols, respectively, as described above.

### CRISPR inhibition

A sgRNA targeting the XIST promoter was designed using the web-based tool CRISPOR (http://crispor.tefor.net/) and cloned into the PB_rtTA_BsmBI vector (a gift from Mauro Calabrese; Addgene 126028)^46^. The sgRNA sequence can be found in Supplementary Table 9. Using the Amaxa 4D-Nucleofector system (Lonza), one million primed H9 hES cells were transfected with 1.5 µg of PB_tre_dCas9_KRAB (a gift from Mauro Calabrese; Addgene 126030), 0.75 µg of PB_rtTA_BsmBI and 1.5 µg of piggyBac transposase^47^. Cells were then treated with G418 (350 µg ml^−1^) and hygromycin (350 µg ml^−1^) until separate colonies were obtained. The number of random insertions in the genome was verified by qPCR and clones with the lowest insertion number (*n* = 2) were used for further experiments. For XIST depletion, PXGL-reprogrammed CRIPSRi naive hES cells were treated with 1 µg ml^−1^ Dox for 10 days.

### CUT&RUN

CUT&RUN was performed as previously described^48^. Briefly, 0.3 million cells per replicate were bound to 20 µl of concanavalin A-coated beads (Bangs Laboratories) in binding buffer (20 mM HEPES, 10 mM KCl, 1 mM CaCl_2_ and 1 mM MnCl_2_). The beads were washed and resuspended in DIG Wash buffer (20 mM HEPES, 150 mM NaCl, 0.5 mM spermidine and 0.05% digitonin). The primary antibodies (1:50) were added to the bead slurry and rotated at room temperature for 1 h. The beads were washed with DIG Wash buffer and protein A–MNase (micrococcal nuclease) fusion protein (1:400, produced by the Institut Curie Recombinant Protein Platform, 0.785 mg ml^−1^) was added, before rotating at room temperature for 15 min. After two washes, the beads were resuspended in 150 µl of DIG Wash buffer and the MNase was activated with 2 mM CaCl_2_, before incubating for 30 min at 0 °C. MNase activity was terminated with 150 µl of 2× Stop buffer (200 mM NaCl, 20 mM EDTA, 4 mM EGTA, 50 µg ml^−1^ RNase A and 40 µg ml^−1^ glycogen). Cleaved DNA fragments were released by incubating for 20 min at 37 °C, followed by centrifugation for 5 min at 16,000*g* at 4 °C and collection of the supernatant from the beads on a magnetic rack. The DNA was purified by phenol–chloroform; libraries were prepared using the TruSeq ChIP Library Preparation Kit from Illumina following the manufacturer’s protocol and sequencing was performed on a NovaSeq 6000 instrument (ICGex next-generation sequencing (NGS) platform) to generate 2 × 100 paired-end reads. All the antibodies used in this study are listed in Supplementary Table 11.

### RAPseq

RAPseq for human XIST was performed as previously described^40^. The human XIST oligo pool was ordered from GenScript and amplified as previously described to generate single-stranded DNA (ssDNA) biotinylated oligos^49^. The oligo sequences are listed in Supplementary Table 12. Briefly, 20 million cells were harvested and incubated with 10 ml of freshly made 2 mM DSG at room temperature for 45 min. Cells were further cross-linked with 10 m of 3% formaldehyde for 10 min at 37 °C and the reaction was stopped by adding glycine to a final concentration of 500 mM. Cells were pelleted at 4 °C and stored at 80 °C. Nuclei were isolated in cold lysis buffer (20 mM HEPES pH 7.5, 50 mM KCl, 1.5 mM MnCl_2_, 1% IGEPAL CA-630 (NP-40), 0.4% sodium deoxycholate and 0.1% *N*-lauroylsarcosine). Chromatin was solubilized by sonication and fragmented by TURBO DNase digestion (Thermo Fisher Scientific). XIST pulldown was performed on 10 million cells using 1 µg of Dynabeads MyOne Streptavidin C1 (Thermo Fisher Scientific) and 50 pmol of biotinylated oligos. DNA was eluted by RNase H digestion and cross-linking was reversed by proteinase K digestion at 65 °C for 1 h. Finally, DNA was purified using the GeneJET Gel Extraction kit (Thermo Fisher Scientific); libraries were prepared using the TruSeq ChIP Library Preparation Kit from Illumina and sequencing was performed on a NovaSeq 6000 instrument (ICGex NGS platform) to generate 2 × 100 paired-end reads.

### Assay for transposase-accessible chromatin with sequencing (ATAC-seq)

ATAC-seq was performed as previously described^50^. Briefly, 50,000 cells were resuspended in 50 µl of cold lysis buffer (10 mM Tris–HCl pH 7.4, 10 mM NaCl, 3 mM MgCl_2_ and 0.1% IGEPAL CA-630) and centrifuged for 10 min at 500*g* at 4 °C. The nuclear pellets were resuspended in 50 μl of transposase reaction mix (25 µl of 2× TD buffer, 2.5 µl of transposase and 22.5 µl of H_2_O) and incubated at 37 °C for 30 min in a ThermoMixer at 1,000 r.p.m. Reactions were cleaned up using the MinElute PCR purification kit (QIAGEN) and DNA was eluted in 10 µl of elution buffer. Transposed DNA was preamplified for five cycles in 50 µl of reaction mix (2.5 μl of 25 μM primer Ad1, 2.5 μl of 25 μM primer Ad2, 25 μl of 2× Master Mix, 10 µl of H_2_O and 10 μl of transposed elution) with the following cycling conditions: 5 min at 72 °C, 30 s at 98 °C and five cycles of 10 s at 98 °C, 30 s at 63 °C and 1 min at 72 °C. Then, 15 μl of qPCR amplification reaction (5 μl of preamplified sample, 0.5 μl of 25 μM primer Ad1, 0.5 μl of 25 μM primer Ad2, 5 μl of 2× NEBNext Master Mix, 0.24 μl of 25× SYBR Green in DMSO and 3.76 μl of H_2_O) was carried out with the following cycling conditions: 30 s at 98 °C and 20 cycles of 10 s at 98 °C, 30 s at 63 °C and 1 min at 72 °C. The required number of additional cycles for each sample was calculated. After the final amplification, double-sided bead purification was performed with AMPure XP beads. Final ATAC-seq libraries were eluted in 20 μl of nuclease-free H_2_O from the beads and were sequenced on a NovaSeq 6000 instrument (Novogene) to generate 2 × 150 paired-end reads.

### RNA-seq

Total RNAs were collected using an RNeasy Mini Kit (QIAGEN) and extracted following the manufacturer’s recommendation. Libraries were generated using the Illumina Stranded Total RNA Prep Ligation with the Ribo-Zero Plus kit according to the manufacturer’s recommendation and sequenced on a NovaSeq 6000 instrument (ICGex NGS platform) to generate 2 × 100 paired-end reads.

### fastGRO-seq

Low-input fastGRO-seq was performed as previously described^28^. Nuclei were isolated from five million cells and a nuclear run-on assay was performed by adding 25 µl of 2× nuclear run-on buffer (10 mM Tris–HCl pH 8, 5 mM MgCl_2_, 300 mM KCl, 1 mM DTT, 500 μM ATP, 500 μM GTP, 500 μM 4-thio-UTP, 2 μM CTP, 200 μg ml^−1^ SUPERase-In and 1% Sarkosyl) at 30 °C for 7 min. RNA was extracted with TRIzol LS reagent (Invitrogen) and quantified using the NanoDrop 2000. A total of 30 µg of RNA was fragmented by sonication and the efficiency was analyzed using the Agilent High-Sensitivity RNA ScreenTape Assay. Fragmented RNA was incubated in biotinylation solution (25 mM HEPES pH 7.4, 10 mM EDTA pH 8.0 and 50 µg of MTS-biotin (Biotium)) for 30 min in the dark at 24 °C and 800 r.p.m. RNA was then precipitated using ethanol and resuspended in nuclease-free water. After DNase treatment, biotinylated RNA was enriched using M280 Streptavidin Dynabeads (Invitrogen) and precipitated using ethanol. Libraries were prepared using the Illumina Stranded Total RNA Prep Ligation with the Ribo-Zero Plus kit according to the manufacturer’s recommendation and sequencing was performed on a NovaSeq 6000 instrument (ICGex NGS platform) to generate 2 × 100 paired-end reads.

### MeD-seq

MeD-seq assays were essentially performed as previously described^51^. Briefly, 10 µl of genomic DNA (input 90 ng) from naive hES cells was digested with LpnPI (New England Biolabs) generating 32-bp fragments around the fully methylated recognition site containing a CpG. These short DNA fragments were further processed using the ThruPLEX DNA-seq 96D Kit (Rubicon Genomics). Stem-loop adaptors were blunt-end ligated to repaired input DNA and amplified to include dual-indexed barcodes using a high-fidelity polymerase to generate an indexed Illumina NGS library. The amplified end product was purified on a Pippin HT system with 3% agarose gel cassettes (Sage Science). Multiplexed samples were sequenced on Illumina NextSeq 2000 systems for single-end reads of 50 bp according to the manufacturer’s instructions. Dual-indexed samples were demultiplexed using bcl2fastq software (Illumina).

### RNA FISH

Cell preparation: Primed hES cells were grown on coverslips. Naive hES cells were centrifuged onto Superfrost Plus slides (VWR) using the Cytospin 3 Cytocentrifuge (Shandon). The cells were fixed for 10 min in a 3% paraformaldehyde solution (Electron Microscopy Science) and permeabilized for 5–10 min in ice-cold cytoskeleton (CSK) buffer (10 mM PIPES, 300 mM sucrose, 100 mM NaCl and 3 mM MgCl_2_ pH 6.8) supplemented with 0.5% Triton X-100 (Sigma-Aldrich) and 2 mM vanadyl ribonucleoside complex (VRC; New England Biolabs).

Probe preparation: RNA FISH probes were obtained after Nick translation of fosmids and BAC constructs purified using phenol–chloroform. Then, 1 μg of purified DNA was labeled for 3 h at 15 °C with fluorescent dUTPs (SpectrumOrange and SpectrumGreen from Abott Molecular and Cy5-UTPs from GE HealthCare Life Science).The templates used in this study were as follows human XIST fosmid (BacPac Resources Center, WI2-3059D20), human *POLA1* BAC (BacPac Resources Center, RP11-11104L9), human XACT BAC (BacPac Resources Center, RP11135D3) and human *HUWE1* BAC (BacPac Resources Center, RP11-975N19).

Hybridization: First, 100 ng of probes were supplemented with 1 μg of Cot-I DNA (Invitrogen) and 3 μg of Sheared Salmon Sperm DNA (Invitrogen). After precipitation, the probes were resuspended in deionized formamide (Sigma-Aldrich), denatured for 7 min at 75 °C and further incubated for 10 min at 37 °C. Probes were mixed with an equal volume of 2× hybridization buffer (4× SSC, 20% dextran sulfate, 2 mg ml^−1^ BSA and 2 mM VRC). Coverslips were dehydrated in 80–100% ethanol washes and incubated with the hybridization mix at 37 °C overnight in a humid chamber. Next, the coverslips were washed for 4 min at 42 °C three times with 50% formaldehyde and 2× SSC (pH 7.2) and three times with 2× SSC. The coverslips were mounted in VECTASHIELD PLUS DAPI (Vector Laboratories).

### Immunofluorescence staining

Primed and naive hES cells were prepared as described above and fixed for 10 min in a 3% paraformaldehyde solution (Electron Microscopy Science). Cells were then permeabilized for 7 min with ice-cold PBS supplemented with 0.5% Triton X-100 (Sigma-Aldrich). Cells were blocked in 1× PBS with 1% BSA (Sigma-Aldrich) for 15 min at room temperature and incubated for 45 min at room temperature with primary antibody diluted in 1× PBS with 1% BSA. After three PBS washes, cells were incubated for 40 min at room temperature with Alexa Fluor 488 anti-rabbit or Alexa Fluor 594 anti-mouse secondary antibodies (Life Technologies). Finally, coverslips were mounted in VECTASHIELD PLUS DAPI (Vector Laboratories). For combined Immunofluorescence and RNA FISH, immunofluorescence staining was first performed and then RNA FISH was performed as described above.

Human embryos were fixed at the B3, B4 or B5 stages according to the grading system proposed by Gardner and Schoolcraft^52^. Embryos were fixed with 4% paraformaldehyde (Electron Microscopy Sciences) for 5 min at room temperature and washed in 1× PBS with 0.1% BSA. Embryos were permeabilized and blocked in 1× PBS with 0.2% Triton and 10% FBS at room temperature for 60 min. Samples were incubated overnight at 4 °C with primary antibodies. After three PBS washes, embryos were incubated for 2 h at room temperature with Alexa Fluor 488 anti-mouse or Alexa Fluor 568 anti-mouse and Alexa Fluor 647 anti-goat secondary antibodies (Life Technologies) along with DAPI counterstaining (Invitrogen). All primary antibodies used in this study are listed in Supplementary Table 11.

### Microscopy and image analysis

Fluorescent microscopy images for hES cells were taken on a fluorescence DMI-6000 inverted microscope with a motorized stage (Leica), equipped with a charge-coupled device (CCD) HQ2 camera (Roper Scientifics) and an HCX PL APO ×64–100 oil objective (numerical aperture, 1.4; Leica) using the MetaMorph software (version 7.04, Roper Scientifics). Approximately 40 optical *z*-sections were collected at 0.3-μm steps at different wavelengths depending on the signal (DAPI, 360–470 nm; FITC, 470–525 nm; Cy3, 550–570 nm; Cy5, 647–668 nm). We computed the dispersion of XIST RNA by comparing the cumulative volume of the signal to the theoretical spherical volume it could occupy on the basis of the maximal radial distance. Embryo confocal immunofluorescence images were acquired with an A1-SIM Nikon confocal microscope and a ×20 oil objective. Whole embryos were imaged with 0.5–1-μm optical sections. Stacks were processed using ImageJ and are represented as a two-dimensional ‘maximum projection’ throughout the manuscript.

### Total RNA extraction and RT–qPCR

Total RNAs were collected using the RNeasy Mini Kit (QIAGEN) and extracted following the manufacturer’s recommendation. Quantification of the extracted RNAs was performed using the NanoDrop 2000. The DNase step was performed on 1 μg of RNA for 30 min at 37 °C using TURBO DNase (Thermo Fisher Scientific). RNAs were reverse-transcribed using the SuperScript IV kit (Thermo Fisher Scientific) following the manufacturer’s recommendation. Complementary DNAs (cDNAs) were diluted 2.5 times in water and the RNA expression level was assessed by RT–qPCR using the Power SYBR Green Master Mix (Thermo Fisher Scientific) and ViiA-7 Real-Time PCR system (Applied Biosystems). Transcript RNA levels were normalized against the β-actin reference gene following the 2^−ΔΔCt^ method. All RT–qPCR primers used in this study are listed in Supplementary Table 13.

### RIP

The cellular extract from 1 million naive H9 hES cells was prepared by adding 1 ml of HNTG buffer (20 mM HEPES pH 7.9, 50 mM NaCl, 1% Triton X-100, 1 mM MgCl_2_, 1 mM EDTA and 10% glycerol) followed by incubation on ice for 25 min. Cells were then centrifuged at 16,000*g* at 4 °C for 25 min. The supernatant was collected and 80 µl was used as the input fraction. Next, 30 μl of Magna ChIP Protein G Magnetic Beads (Merck) per immunoprecipitation were washed with PBS–Tween 0.1% three times. Then, 2.5 μg of SPEN antibody (Abcam, AB72266) was added to the beads, followed by 10-min incubation at room temperature with rotation. Beads were washed three times with PBS–Tween 0.1% and resuspended with HNTG buffer. Next, 30 μl of beads were added to the lysate prepared above and incubated for 1.5 h at 4 °C with rotation. After immunoprecipitation, beads were washed three times with 500 μl of HNTG buffer. For RNA preparation, input and immunoprecipitation samples were treated with 200 μg of proteinase K in a total volume of 200 μl for 45 min at 65 °C. RNA was purified using the RNeasy MinElute Cleanup Kit (Qiagen) according to the manufacturer’s recommendations in a final volume of 14 μl of water. The DNase step and RT were performed as described above. The RNA enrichment level was assessed by RT–qPCR using the Power SYBR Green Master Mix (Thermo Fisher Scientific) and ViiA-7 Real-Time PCR system (Applied Biosystems).

### siRNA KD SPEN

SPEN KD was achieved using two different mixes of siRNAs. Mix 1 contained one siRNA (CliniSciences, CRH7929) and mix 2 contained two siRNAs (Thermo Fisher Scientific, 1299001 and 4392420). As a negative control, scramble siRNA was used (ThermoFisher Scientific, AM4611). Naive H9 cells were transfected using the lipofectamine RNAiMAX transfection reagent (ThermoFisher Scientific, 13778030) according to the manufacturer’s recommendations. PXGL medium was changed 1 h before transfection. After 24 h, another round of transfection was performed. After 48 h in total, cells were collected and RNA was extracted using the RNeasy Mini Kit (Qiagen) according to the manufacturer’s recommendation.

### Raw data processing

RNA-seq, fastGRO-seq, CUT&RUN and RAPseq reads were trimmed using trim_galore (version 0.6.5) (https://github.com/FelixKrueger/TrimGalore) with a minimum length of 50 bp. Reads were then mapped to the human genome (hg38) and mouse genome (mm10) using Bowtie 2 (version 2.4.4)^53^ with the following parameters: ‘--local --very-sensitive-local --no-unal --no-mixed --no-discordant --phred33 -L 10 -X 700’. Reads were then deduplicated using MarkDuplicates from Picard (version 2.23.5) (http://broadinstitute.github.io/picard/) with the following options: ‘--CREATE_INDEX = true --VALIDATION_STRINGENCY = SILENT --REMOVE_DUPLICATES = true --ASSUME_SORTED = true’. BAM files were sorted, filtered (minimum mapping quality = 10) and indexed with SAMtools (version 1.13)^54^. Reads from MEFs were discarded using the XenofilteR package in R (version 4.1.1)^55^. Replicate reproducibility was assessed by Pearson’s correlation of the signal using deepTools plotCorrelation.

### RNA-seq and fastGRO-seq data analysis

For gene expression analysis, read counts were quantified for each gene with htseq-count from htseq (version 0.13.5)^56^ using the following options: ‘--stranded reverse -a 10 -t exon -i gene_id -m intersection-nonempty’. The annotation file used was Homo_sapiens.GRCh38.90.gtf. Reads marked by special counters (no feature, ambiguous, too low aQual, not aligned or alignment not unique) were eliminated. For XIST isoform reconstruction, Scallop (version 0.10.4) was used with the default parameters^57^. XIST isoform quantification was performed using kallisto (version 0.46.2) on the trimmed FASTQ file with the option ‘--rf-stranded’ (ref. ^58^). For that purpose, the transcriptome FASTA file of exons and introns was generated from the hg38 reference genome using the BUSpaRse package in R (version 4.1.1) and used to produce a kallisto index.

### Single-cell expression data analysis

FASTQ files from refs. ^20,25^ were obtained from the ENA (European Nucleotide Archive), under accession numbers PRJEB11202 and PRJNA431392, respectively. STAR (version 2.7.9a) was used for read alignment to the GRCh38 reference human genome. The STAR index was generated using the following options: ‘--runMode genomeGenerate --runThreadN 30 --genomeFastaFiles HS.GRCh38.fa --limitGenomeGenerateRAM 168632718037 --outFileNamePrefix STAR_INDEX. --sjdbGTFfile Homo_sapiens.GRCh38.90.gtf --genomeDir STAR_INDEX_petropoulos_2016 --sjdbOverhang 42’ for Petropoulos data and ‘--genomeDir STAR_INDEX --sjdbOverhang 149’ for Zhou data.

For Petropoulos data, reads (single-end) were aligned using the following options: ‘--soloType SmartSeq --soloFeatures Gene --runThreadN 12 --genomeDir STAR_INDEX_petropoulos_2016 --readFilesCommand zcat --readFilesIn sample_A.fastq.gz --soloStrand Unstranded --soloUMIdedup NoDedup --outFileNamePrefix petro_2016/STAR_ALIGN_sample_A.STARSOLO --outSAMtype BAM SortedByCoordinate --outSAMattrRGline ID:sample_A’.

For Zhou data reads (paired-end) were aligned using the following options: ‘--soloType CB_UMI_Simple --soloCBwhitelist zhou_2019/whitelist.txt --soloFeatures Gene Velocyto --runThreadN 5 --genomeDir STAR_INDEX --readFilesCommand zcat --readFilesIn multiplex_sample_A_R1.fastq.gz multiplex_sample_A_R2.fastq.gz --outFileNamePrefix zhou_2019/STAR_ALIGN_multiplex_sample_A.STARSOLO --outSAMtype BAM SortedByCoordinate --outSAMattributes NH HI nM AS CR UR CB UB GX GN sS sQ sM --soloCBstart 1 --soloCBlen 8 --soloUMIstart 9 --soloUMIlen 7 --soloCellFilter None --soloBarcodeReadLength 0’.

For female embryos, single cells were grouped by cell type and developmental day, according to the published sample annotation^20,25^ refined by clustering on the UMAP (uniform manifold approximation and projection) of batch-corrected data (‘3_buildModel.R’ in https://gitlab.univ-nantes.fr/E137833T/Castel_et_al_2020/-/blob/master/Code/). The sum of raw counts and counts per million (CPM) normalization were computed for each group. The median gene expression of the X chromosome and autosomes was calculated after removing nondetected genes.

### SNP calling

We used a whole-genome sequencing dataset of H9 hES cells to identify informative genomic SNPs along the X chromosome that can be used for allelic expression analysis (GSM1227088). Reads were aligned using Bowtie 2 and PCR duplicates were filtered out using MarkDuplicates from Picard (version 2.22.0) as previously described. For proper tag formatting of BAM files, AddorReplaceReadGroups from Picard was used with the following options: ‘SO = coordinate, RGID = id, RGLB = library, RGPL = platform, RGPU = machine, RGSM = sample’. BAM files were further processed using GATK (version 4.1.2.0)^59^ for SNP identification. BaseRecalibrator and ApplyBQSR were used to generate the recalibrated BAM file using high-confidence SNPs referenced in Mills_and_1000G_gold_standard.indels.hg38.vcf.gz (annotation of indels) and dbsnp_146.hg38.vcf.gz (annotation of SNPs). The Variant Call Format (VCF) file H9_WGS_hg38_filtered.vcf was produced with HaplotypeCaller (options: ‘--dont-use-soft-clipped-bases and -stand-call-conf 10.0’) and filtered using VariantFiltration (options: ‘-window 50 -cluster 3 --filter-name FS -filter FS > 30.0 --filter-name QD -filter QD < 2.0’). The SNP position file info_snp_heterozygous_h9_wgs_chrX.txt was produced from VCF in R (version 4.1.1), using minimum thresholds of 50 for quality score, 10 for read coverage and 0.25 for allelic ratio to define heterozygous positions. We then used mpileup from SAMtools (version 1.15.1) to produce pileup files from RNA-seq and fastGRO-seq BAM files with the following options: ‘--output-MQ --no-output-ins --no-output-ins --no-output-del --no-output-del --no-output-ends’. The putative Xa haplotype was generated in R (version 4.1.1) from monoallelically expressed SNPs from the X chromosome in H9 primed hES cells. This was used to determine the pXi:Xa allelic ratio across all samples. We considered a transcript as biallelic when at least 25% of reads originated from the second allele.

### XIST-sensitive and XIST-resistant gene calling

We applied stringent criteria for defining genes sensitive or resistant to XIST in naive cells. First, we determined the pXi:Xa allelic ratio for all detected SNPs, as described above, in XIST KO and CRISPRi datasets. We then computed the FC of the pXi:Xa allelic ratio between the XIST KO and WT and the XIST Dox-treated and UT CRISPRi datasets. An X-linked gene was defined as sensitive to XIST when the FC was greater than 1.2 in both datasets.

### CUT&RUN data analysis

BigWig track files were generated with bamCoverage from deepTools (version 3.5.0) using the following parameters: ‘--normalizeUsing BPM --binSize 20 --smoothLength 40’. Coverage score matrices were generated with computeMatrix from deepTools (version 3.5.0) using either reference-point (‘--referencePoint TSS -R Homo_sapiens.GRCh38.90.gtf --beforeRegionStartLength 1000 --afterRegionStartLength 1000 --binSize 20 --missingDataAsZero’) or scale-regions (‘-R Homo_sapiens.GRCh38.90.gtf --regionBodyLength 20000 --startLabel TSS --endLabel TES --binSize 20 --missingDataAsZero’). For histone modification enrichment, raw counts were generated using multiBigwigSummary from deepTools with ‘--binSize 10000’ and the mean counts per chromosome were calculated. The percentage occupancy was determined by calculating for each chromosome the cumulative coverage over the chromosome size. Regions with a minimum threshold of 1.2 (FC over IgG) were considered covered.

### ATAC-seq data analysis

ATAC-seq raw data were analyzed using the atacseq pipeline (version 1.2.1) (https://github.com/nf-core/atacseq/) from nf-core^60^ using the default parameters. Briefly, reads were aligned to the human genome (hg38) using BWA (version 0.7.17). Duplicates were marked by Picard and reads mapping to mitochondrial DNA and blacklisted regions were removed. BigWig files were generated using deepTools as described above.

### MeD-seq data analysis

Data processing was carried out using specifically created scripts in Python. Raw FASTQ files were subjected to Illumina adaptor trimming and reads were filtered on the basis of LpnPI restriction site occurrence 13–17 bp from either the 5′ or 3′ end of the read. Reads that passed the filter were mapped to hg38 using Bowtie 2. Genome-wide individual LpnPI site scores were used to generate read count scores for the following annotated regions: TSSs (1 kb before and 1 kb after), CpG islands and gene bodies (1 kb after TSS until TES (transcription end site)) and normalized (reads per million mapped reads (RPM)) using the total number of CpG reads after the filter. Gene and CpG island annotations were downloaded from ENSEMBL (www.ensembl.org).

### RAPseq data analysis

BigWig tracks were generated as previously described. RAPseq naive versus primed log_2_ enrichment was calculated using bigwigCompare from deepTools with the default parameters. The percentage occupancy was determined by calculating for each chromosome the cumulative coverage over the chromosome size. Regions with a minimum threshold of 10 (FC over input) were considered covered. RAPseq coverage scores were calculated with multiBigwigSummary, using the bins option for gene density enrichment and BED-file option for TEs (annotation from RepeatMasker).

### Statistics and reproducibility

Statistical analyses were performed in R (version 4.1.1). The statistical analysis method for each experiment is specified in the corresponding figure legend. *P* values < 0.05 were considered statistically significant. Unless otherwise mentioned, most of the data shown are either representative of three or more independent experiments or combined from three or more biologically independent samples and analyzed as the mean ± s.d.

### Reporting summary

Further information on research design is available in the Nature Portfolio Reporting Summary linked to this article.

## Online content

Any methods, additional references, Nature Portfolio reporting summaries, source data, extended data, supplementary information, acknowledgements, peer review information; details of author contributions and competing interests; and statements of data and code availability are available at 10.1038/s41594-024-01325-3.

## Supplementary information

Reporting Summary

Supplementary Table 1 List of informative SNPs on chromosomes X and 7 used to determine the percentage of biallelism.

Supplementary Tables 2–13 **Supplementary Table 2** List of DEGs upon XIST KO in naive H9 cells. **Supplementary Table 3** List of DEGs upon XIST repression by Dox treatment in naive H9 cells. **Supplementary Table 4** List of DEGs upon XACT KO in naive H9 cells. **Supplementary Table 5** pXi allelic ratio of XIST-sensitive and XIST-resistant X-linked genes in naive H9 cells. **Supplementary Table 6** List of DEGs upon SPEN KD (siSPEN1 versus siScr) in naive H9 cells. **Supplementary Table 7** List of DEGs upon SPEN KD (siSPEN2 versus siScr) in naive H9 cells. **Supplementary Table 8** pXi allelic ratio of XIST-sensitive and XIST-resistant X-linked genes upon SPEN KD in naive H9 cells. **Supplementary Table 9** Sequences of the sgRNAs used in this study. **Supplementary Table 10** Sequences of primers for PCR genotyping used in this study. **Supplementary Table 11** List of antibodies used in this study. **Supplementary Table 12** Sequences of XIST RAPseq oligos used in this study. **Supplementary Table 13** Sequences of RT–qPCR primers used in this study.

## Source data

Source Data Fig. 1 Raw data for graphs.

Source Data Fig. 2 Raw data for graphs.

Source Data Fig. 3 Raw data for graphs.

Source Data Fig. 4 Raw data for graphs.

Source Data Fig. 5 Raw data for graphs.

Source Data Fig. 6 Raw data for graphs.

Source Data Extended Data Fig. 1 and Table 1 Raw data for graphs.

Source Data Extended Data Fig. 2 and Table 2 Raw data for graphs.

Source Data Extended Data Fig. 3 and Table 3 Raw data for graphs.

Source Data Extended Data Fig. 4 and Table 4 Raw data for graphs.

Source Data Extended Data Fig. 5 and Table 5 Raw data for graphs.

Source Data Extended Data Fig. 6 and Table 6 Raw data for graphs.

## Data Availability

All RNA-seq, RAPseq, ATAC-seq, CUT&RUN, MeD-seq and fastGRO-seq data were deposited on the Gene Expression Omnibus (GSE246643). Microscopy data reported in this paper will be shared by the corresponding author upon request. This paper analyzed existing, publicly available data. scRNA-seq data from human embryos are available from the ENA under accession numbers PRJEB11202 and PRJNA431392. CUT&RUN and Minute ChIP data for naive H9 hES cells are available at GSE176175 and GSE181244, respectively. Source data are provided with this paper.

## Code availability

All original code was deposited on GitHub (https://github.com/gaelcastel/charbel_paper_github_v1.git)^61^ and is publicly available as of the date of publication. Any additional information required to reanalyze the data reported in this paper is available from the corresponding author upon request.
