# Supplementary material for: XIST dampens X chromosome activity in a SPEN-dependent manner during early human development
Source: Nat Struct Mol Biol. 2024 Jun 4;31(10):1589–600. doi: 10.1038/s41594-024-01325-3 (PMC11479943; doi:10.1038/s41594-024-01325-3)
Supplement: Supplementary file 1 — Reporting Summary [file 41594_2024_1325_MOESM1_ESM.pdf]

nature portfolio

Corresponding author(s): Claire Rougeulle

Last updated by author(s): Apr 2, 2024

Reporting Summary

Nature Portfolio wishes to improve the reproducibility of the work that we publish. This form provides structure for consistency and transparency in reporting. For further information on Nature Portfolio policies, see our Editorial Policies and the Editorial Policy Checklist.

Statistics

For all statistical analyses, confirm that the following items are present in the figure legend, table legend, main text, or Methods section.

n/a	Confirmed	
☐	☒	The exact sample size (*n*) for each experimental group/condition, given as a discrete number and unit of measurement
☐	☒	A statement on whether measurements were taken from distinct samples or whether the same sample was measured repeatedly
☐	☒	The statistical test(s) used AND whether they are one- or two-sided<br>*Only common tests should be described solely by name; describe more complex techniques in the Methods section.*
☒	☐	A description of all covariates tested
☐	☒	A description of any assumptions or corrections, such as tests of normality and adjustment for multiple comparisons
☐	☒	A full description of the statistical parameters including central tendency (e.g. means) or other basic estimates (e.g. regression coefficient) AND variation (e.g. standard deviation) or associated estimates of uncertainty (e.g. confidence intervals)
☐	☒	For null hypothesis testing, the test statistic (e.g. *F*, *t*, *r*) with confidence intervals, effect sizes, degrees of freedom and *P* value noted<br>*Give P values as exact values whenever suitable.*
☒	☐	For Bayesian analysis, information on the choice of priors and Markov chain Monte Carlo settings
☒	☐	For hierarchical and complex designs, identification of the appropriate level for tests and full reporting of outcomes
☐	☒	Estimates of effect sizes (e.g. Cohen's *d*, Pearson's *r*), indicating how they were calculated

Our web collection on statistics for biologists contains articles on many of the points above.

Software and code

Policy information about availability of computer code

Data collection	For CUT&RUN, RAP-seq, RNA-seq, and fastGRO-seq data collection, illumina Novaseq 6000 platform with paired-end settings was used. For MeD-seq, Illumina NextSeq2000 platform with single-end settings was used. More information can be found in the methods section of the manuscript.
Data analysis	R (4.1.1)<br><br>trim_galore (0.6.5)<br>bowtie2 (2.4.4)<br>picard (2.23.5)<br>samtools (1.13)<br>deeptools (3.5.0)<br>htseq (0.13.5)<br>Scallop (0.10.4)<br>kallisto (0.46.2)<br>GATK (4.1.2.0)<br>atacseq pipeline from nf-core (1.2.1)<br>BWA (0.7.17)<br>Fiji v2.0.0<br>STAR (2.7.9a)<br><br>Metamorph software (version 7.04, Roper Scientifics)

All original code has been deposited at GitHub (https://github.com/gaelcastel/charbel_paper_github_v1) and is publicly available as of the date of publication.

For manuscripts utilizing custom algorithms or software that are central to the research but not yet described in published literature, software must be made available to editors and reviewers. We strongly encourage code deposition in a community repository (e.g. GitHub). See the Nature Portfolio guidelines for submitting code & software for further information.

Data

Policy information about availability of data

All manuscripts must include a data availability statement. This statement should provide the following information, where applicable:

- Accession codes, unique identifiers, or web links for publicly available datasets
- A description of any restrictions on data availability
- For clinical datasets or third party data, please ensure that the statement adheres to our policy

All RNA-seq, RAP-seq, ATAC-seq, CUT&RUN, MeD-seq, and fastGRO-seq data have been deposited at GEO (GSE246643) and are publicly available as of the date of publication.

Microscopy data reported in this paper will be shared by the lead contact upon request.

This paper analyzes existing, publicly available data. scRNA-seq data from human embryos are available at ENA (European Nucleotide Archive), under accession numbers PRJEB11202 and PRJNA431392. CUT&RUN and Minute ChIP data for naïve H9 hESCs are available at GSE176175 and GSE181244, respectively.

Research involving human participants, their data, or biological material

Policy information about studies with human participants or human data. See also policy information about sex, gender (identity/presentation), and sexual orientation and race, ethnicity and racism.

Reporting on sex and gender	*Use the terms sex (biological attribute) and gender (shaped by social and cultural circumstances) carefully in order to avoid confusing both terms. Indicate if findings apply to only one sex or gender; describe whether sex and gender were considered in study design; whether sex and/or gender was determined based on self-reporting or assigned and methods used. Provide in the source data disaggregated sex and gender data, where this information has been collected, and if consent has been obtained for sharing of individual-level data; provide overall numbers in this Reporting Summary. Please state if this information has not been collected. Report sex- and gender-based analyses where performed, justify reasons for lack of sex- and gender-based analysis.*
Reporting on race, ethnicity, or other socially relevant groupings	*Please specify the socially constructed or socially relevant categorization variable(s) used in your manuscript and explain why they were used. Please note that such variables should not be used as proxies for other socially constructed/relevant variables (for example, race or ethnicity should not be used as a proxy for socioeconomic status). Provide clear definitions of the relevant terms used, how they were provided (by the participants/respondents, the researchers, or third parties), and the method(s) used to classify people into the different categories (e.g. self-report, census or administrative data, social media data, etc.) Please provide details about how you controlled for confounding variables in your analyses.*
Population characteristics	*Describe the covariate-relevant population characteristics of the human research participants (e.g. age, genotypic information, past and current diagnosis and treatment categories). If you filled out the behavioural & social sciences study design questions and have nothing to add here, write "See above."*
Recruitment	*Describe how participants were recruited. Outline any potential self-selection bias or other biases that may be present and how these are likely to impact results.*
Ethics oversight	*Identify the organization(s) that approved the study protocol.*

Note that full information on the approval of the study protocol must also be provided in the manuscript.

Field-specific reporting

Please select the one below that is the best fit for your research. If you are not sure, read the appropriate sections before making your selection.

☒ Life sciences ☐ Behavioural & social sciences ☐ Ecological, evolutionary & environmental sciences

For a reference copy of the document with all sections, see nature.com/documents/nr-reporting-summary-flat.pdf

Life sciences study design

All studies must disclose on these points even when the disclosure is negative.

Sample size	Sample size for omics experiments:<br>2 biological replicates for all CUT&RUN experiments except for XIST CRISPRi untreated and Dox-treated CUT&RUN in which 3 biological replicates were sequenced.

	2 biological replicates for RAP-seq.<br>3 biological replicates for MeD-seq and ATAC-seq.<br>3 biological replicates for RNA-seq and fastGRO-seq except for XIST KO RNA-seq in which 3 independent technical replicates were sequenced.
Data exclusions	For all omics data generated from H9 naive hESCs, reads from mouse embryonic fibroblasts (MEFs) were discarded using XenofilteR package from R (4.1.1).<br>For RNA-seq and fastGRO-seq data analysis, reads marked by special counters (no feature, ambiguous, too low aQual, not aligned, alignment not unique) were eliminated.<br>For SNP calling, SNPs with minimum threshold 50 for quality score and 10 for read coverage were considered.
Replication	Unless otherwise mentioned, all of the main experiments listed on this manuscript have been successfully replicated at least three times as independent experiments.
Randomization	Sample allocation was random for all experiments.
Blinding	Samples from naive vs primed H9, XIST - vs XIST +, XACT KO vs WT were used and hence the analyses could not be performed with blinding.<br>No blinding was performed as none of the reported analyses involved procedures that could be influenced by investigator bias.

Reporting for specific materials, systems and methods

We require information from authors about some types of materials, experimental systems and methods used in many studies. Here, indicate whether each material, system or method listed is relevant to your study. If you are not sure if a list item applies to your research, read the appropriate section before selecting a response.

Materials & experimental systems

n/a	Involved in the study
☐	☒ Antibodies
☐	☒ Eukaryotic cell lines
☒	☐ Palaeontology and archaeology
☒	☐ Animals and other organisms
☒	☐ Clinical data
☒	☐ Dual use research of concern
☒	☐ Plants

Methods

n/a	Involved in the study
☐	☒ ChIP-seq
☒	☐ Flow cytometry
☒	☐ MRI-based neuroimaging

Antibodies

Antibodies used	Rabbit polyclonal anti-H3K4me3, Active motif, Cat#39060, CUT&RUN, 1:50<br>Rabbit polyclonal anti-H3K9me3, Active motif, Cat#39062, CUT&RUN, 1:50<br>Rabbit polyclonal anti-H3K27me3, Active motif, Cat#39055, CUT&RUN, 1:50<br>Rabbit monoclonal anti-H2AK119Ub (D27C4), Cell Signaling Technology, Cat#8240S, CUT&RUN and IF hESCs 1:50 and 1/800 respectively<br>Rabbit polyclonal anti-IgG, Diagenode, Cat#C15410206, CUT&RUN, 1:50<br>Rabbit polyclonal anti-H3K27ac, Active motif, Cat#39034, CUT&RUN, 1:50<br>Mouse polyclonal anti-H3K27me3, Active motif, Cat# 61018, IF hESCs, 1/500<br>Goat polyclonal anti-NANOG, R&D systems, AF1997, IF human embryos, 1/300<br>Mouse monoclonal anti-H3K27me3, Abcam, AB6002, IF human embryos, 1/300<br>Rabbit polyclonal anti-SPEN, Abcam, AB72266, RIP, 2.5µg
Validation	All antibodies were previously validated by vendors and/or published work.<br><br>Antibodies from Active motif were validated for CUT&RUN https://www.activemotif.com/catalog/1375/cut-run-validated-antibodies#:~:text=CUT%26RUN%20is%20a%20derivative%20of%20chromatin%20immunocleavage%20%28ChIC%29.,sample%20material%20and%20less%20sequencing%20depths%20than%20ChIP.<br><br>Rabbit monoclonal anti-H2AK119Ub, Cell Signaling Technology, Cat#8240S, https://www.cellsignal.com/products/primary-antibodies/ubiquityl-histone-h2a-lys119-d27c4-xp-rabbit-mab/8240, cited in 310 publications.<br><br>Goat polyclonal anti-NANOG, R&D systems, AF1997, hhttps://www.rndsystems.com/products/human-nanog-antibody_af1997, cited in 207 publications.<br><br>Mouse monoclonal anti-H3K27me3, Abcam, AB6002, https://www.abcam.com/products/primary-antibodies/histone-h3-tri-methyl-k27-antibody-mabcam-6002-chip-grade-ab6002.html, cited in 908 publications.<br><br>Rabbit polyclonal anti-SPEN, Abcam, AB72266, https://www.abcam.com/products/primary-antibodies/spen-antibody-ab72266.html, cited in 3 publications.<br><br>Rabbit polyclonal anti-IgG, Diagenode, Cat#C15410206, https://www.diagenode.com/en/p/rabbit-igg-250-ug-250-ul#

Eukaryotic cell lines

Policy information about cell lines and Sex and Gender in Research

Cell line source(s)	H9 hESCs (WiCell)
Authentication	Cell lines were not further authenticated.
Mycoplasma contamination	All cell lines were routinely tested for mycoplasma contamination.
Commonly misidentified lines (See ICLAC register)	No cell line used in this study is listed in the ICLAC database.

Plants

Seed stocks	*Report on the source of all seed stocks or other plant material used. If applicable, state the seed stock centre and catalogue number. If plant specimens were collected from the field, describe the collection location, date and sampling procedures.*
Novel plant genotypes	*Describe the methods by which all novel plant genotypes were produced. This includes those generated by transgenic approaches, gene editing, chemical/radiation-based mutagenesis and hybridization. For transgenic lines, describe the transformation method, the number of independent lines analyzed and the generation upon which experiments were performed. For gene-edited lines, describe the editor used, the endogenous sequence targeted for editing, the targeting guide RNA sequence (if applicable) and how the editor was applied.*
Authentication	*Describe any authentication procedures for each seed stock used or novel genotype generated. Describe any experiments used to assess the effect of a mutation and, where applicable, how potential secondary effects (e.g. second site T-DNA insertions, mosiacism, off-target gene editing) were examined.*

ChIP-seq

Data deposition

☒ Confirm that both raw and final processed data have been deposited in a public database such as GEO.

☒ Confirm that you have deposited or provided access to graph files (e.g. BED files) for the called peaks.

Data access links *May remain private before publication.*	https://www.ncbi.nlm.nih.gov/geo/query/acc.cgi?acc=GSE246643
Files in database submission	GSM7873790 PXGL, H3K27ac, CRISPRi XIST UT, Rep1<br>GSM7873791 PXGL, H3K4me3, CRISPRi XIST UT, Rep1<br>GSM7873792 PXGL, H3K27me3, CRISPRi XIST UT, Rep1<br>GSM7873793 PXGL, H3K9me3, CRISPRi XIST UT, Rep1<br>GSM7873794 PXGL, H2AK119Ub, CRISPRi XIST UT, Rep1<br>GSM7873795 PXGL, IgG, CRISPRi XIST UT, Rep1<br>GSM7873796 PXGL, H3K27ac, CRISPRi XIST UT, Rep2<br>GSM7873797 PXGL, H3K4me3, CRISPRi XIST UT, Rep2<br>GSM7873798 PXGL, H3K27me3, CRISPRi XIST UT, Rep2<br>GSM7873799 PXGL, H3K9me3, CRISPRi XIST UT, Rep2<br>GSM7873800 PXGL, H2AK119Ub, CRISPRi XIST UT, Rep2<br>GSM7873801 PXGL, IgG, CRISPRi XIST UT, Rep2<br>GSM7873802 PXGL, H3K27ac, CRISPRi XIST UT, Rep3<br>GSM7873803 PXGL, H3K4me3, CRISPRi XIST UT, Rep3<br>GSM7873804 PXGL, H3K27me3, CRISPRi XIST UT, Rep3<br>GSM7873805 PXGL, H3K9me3, CRISPRi XIST UT, Rep3<br>GSM7873806 PXGL, H2AK119Ub, CRISPRi XIST UT, Rep3<br>GSM7873807 PXGL, IgG, CRISPRi XIST UT, Rep3<br>GSM7873808 PXGL, H3K27ac, CRISPRi XIST Dox, Rep1<br>GSM7873809 PXGL, H3K4me3, CRISPRi XIST Dox, Rep1<br>GSM7873810 PXGL, H3K27me3, CRISPRi XIST Dox, Rep1<br>GSM7873811 PXGL, H3K9me3, CRISPRi XIST Dox, Rep1<br>GSM7873812 PXGL, H2AK119Ub, CRISPRi XIST Dox, Rep1<br>GSM7873813 PXGL, IgG, CRISPRi XIST Dox, Rep1<br>GSM7873814 PXGL, H3K27ac, CRISPRi XIST Dox, Rep2<br>GSM7873815 PXGL, H3K4me3, CRISPRi XIST Dox, Rep2<br>GSM7873816 PXGL, H3K27me3, CRISPRi XIST Dox, Rep2<br>GSM7873817 PXGL, H3K9me3, CRISPRi XIST Dox, Rep2<br>GSM7873818 PXGL, H2AK119Ub, CRISPRi XIST Dox, Rep2<br>GSM7873819 PXGL, IgG, CRISPRi XIST Dox, Rep2<br>GSM7873820 PXGL, H3K27ac, CRISPRi XIST Dox, Rep3<br>GSM7873821 PXGL, H3K4me3, CRISPRi XIST Dox, Rep3<br>GSM7873822 PXGL, H3K27me3, CRISPRi XIST Dox, Rep3<br>GSM7873823 PXGL, H3K9me3, CRISPRi XIST Dox, Rep3

GSM7873824 PXGL, H2AK119Ub, CRISPRi XIST Dox, Rep3
GSM7873825 PXGL, IgG, CRISPRi XIST Dox, Rep3
GSM7873826 PXGL, H3K27ac, WT, Rep1
GSM7873827 PXGL, H3K4me3, WT, Rep1
GSM7873828 PXGL, H3K27me3, WT, Rep1
GSM7873829 PXGL, H3K9me3, WT, Rep1
GSM7873830 PXGL, H2AK119Ub, WT, Rep1
GSM7873831 PXGL, IgG, WT, Rep1
GSM7873832 PXGL, H3K27ac, KO, Rep1
GSM7873833 PXGL, H3K4me3, KO, Rep1
GSM7873834 PXGL, H3K27me3, KO, Rep1
GSM7873835 PXGL, H3K9me3, KO, Rep1
GSM7873836 PXGL, H2AK119Ub, KO, Rep1
GSM7873837 PXGL, IgG, KO, Rep1
GSM7873838 PXGL, H3K27ac, WT, Rep2
GSM7873839 PXGL, H3K4me3, WT, Rep2
GSM7873840 PXGL, H3K27me3, WT, Rep2
GSM7873841 PXGL, H3K9me3, WT, Rep2
GSM7873842 PXGL, H2AK119Ub, WT, Rep2
GSM7873843 PXGL, IgG, WT, Rep2
GSM7873844 PXGL, H3K27ac, KO, Rep2
GSM7873845 PXGL, H3K4me3, KO, Rep2
GSM7873846 PXGL, H3K27me3, KO, Rep2
GSM7873847 PXGL, H3K9me3, KO, Rep2
GSM7873848 PXGL, H2AK119Ub, KO, Rep2
GSM7873849 PXGL, IgG, KO, Rep2
GSM7873850 Primed, H3K4me3, Rep1
GSM7873851 Primed, H3K27me3, Rep1
GSM7873852 Primed, H3K9me3, Rep1
GSM7873853 Primed, H2AK119Ub, Rep1
GSM7873854 Primed, IgG, Rep1
GSM7873855 Primed, H3K4me3, Rep2
GSM7873856 Primed, H3K27me3, Rep2
GSM7873857 Primed, H3K9me3, Rep2
GSM7873858 Primed, H2AK119Ub, Rep2
GSM7873859 Primed, IgG, Rep2

Genome browser session (e.g. UCSC)

n/a

Methodology

Replicates

Two biological replicates. For XIST CRISPRi clones three biological replicates were sequenced.

Sequencing depth

CUT&RUN replicates were sequenced at a average depth of 15 million paired-end reads.

Antibodies

Rabbit polyclonal anti-H3K4me3, Active motif, Cat#39060, CUT&RUN, 1:50
Rabbit polyclonal anti-H3K9me3, Active motif, Cat#39062, CUT&RUN, 1:50
Rabbit polyclonal anti-H3K27me3, Active motif, Cat#39055, CUT&RUN, 1:50
Rabbit monoclonal anti-H2AK119Ub, Cell Signaling Technology, Cat#8240S, CUT&RUN, 1:50
Rabbit polyclonal anti-H3K27ac, Active motif, Cat#39034, CUT&RUN, 1:50
Rabbit polyclonal anti-IgG, Diagenode, Cat#C15410206, CUT&RUN, 1:50

Peak calling parameters

No peak calling was done.

Data quality

Reads were trimmed using trim_galore with a minimum length of 50 bp. Reads were then mapped to the human genome (hg38) and mouse genome (mm10) using bowtie2 with the following parameters: --local --very-sensitive-local --no-unal --no-mixed --no-discordant --phred33 -L 10 -X 700. Reads were then deduplicated using MarkDuplicates from picard with the following options: --CREATE_INDEX=true --VALIDATION_STRINGENCY=SILENT --REMOVE_DUPLICATES=true --ASSUME_SORTED=true. Bam files were sorted, filtered (minimum mapping quality=10) and indexed with samtools. Reads from mouse embryonic fibroblasts (MEFs) were discarded using XenofilteR package from R.

Software

R (4.1.1)
trim_galore (0.6.5)
bowtie2 (2.4.4)
picard (2.23.5)
samtools (1.13)
deeptools (3.5.0)
